# Cancer Cell‐Derived Large Extracellular Vesicles Promote Venous Thromboembolism by Activating NETosis Through Delivering CYBA

**DOI:** 10.1002/advs.202507867

**Published:** 2025-07-21

**Authors:** Xiangji Li, Yingjiao Ju, Chenjie Xu, Shixiang Ma, Lan Sun, Qingdong Guo, Mingyuan Liu, Yibin Xie, Li Min

**Affiliations:** ^1^ Department of Gastroenterology Beijing Friendship Hospital Capital Medical University State Key Laboratory of Digestive Health National Clinical Research Center for Digestive Diseases Beijing Key Laboratory for Precancerous Lesion of Digestive Diseases Beijing 100050 P. R. China; ^2^ Research Center Beijing Clinical Research Institute Beijing Friendship Hospital Capital Medical University Beijing 100050 P. R. China; ^3^ Department of Retroperitoneal Tumor Surgery Peking University International Hospital Beijing 102206 P. R. China; ^4^ Institute of Basic Research in Clinical Medicine China Academy of Chinese Medical Sciences Beijing 100700 P. R. China; ^5^ Department of Vascular Surgery Beijing Friendship Hospital Capital Medical University Beijing 100050 P. R. China; ^6^ Department of Pancreatic and Gastric Surgery National Cancer Center/National Clinical Research Center for Cancer/Cancer Hospital Chinese Academy of Medical Sciences and Peking Union Medical College Beijing 100021 P. R. China

**Keywords:** cancer, CYBA, extracellular vesicles, NETosis, venous thromboembolism

## Abstract

Venous thromboembolism (VTE) is the second‐leading cause of cancer‐associated mortality. Neutrophil extracellular trap formation (i.e., NETosis) is a crucial process in forming VTE in cancer patients. Nevertheless, how cancer cells contribute to NETosis remains unclear. This study investigated the potential activation effects of cancer cell‐derived extracellular vesicles (CC‐EVs) on neutrophils. Both small and large EVs (sEVs and lEVs) released from cancer cells are found to significantly induce NETosis in neutrophil‐like HL‐60 (dHL‐60) cells. Following an in‐depth exploration of EV‐induced NETosis, the specific molecular pathways involved in this biological process are elucidated. CYBA enriched in CC‐lEVs is delivered to dHL‐60, leading to a rapid increase in intracellular ROS levels and upregulation of citH3 expression. This cascade resulted in decondensed chromatin release and subsequent NETosis along with elevated MPO‐DNA levels. Injection of CC‐lEVs into mice caused more pronounced VTE, which is accompanied by increased peripheral blood levels of the MPO‐DNA and thrombin‐antithrombin complex. Inhibiting CYBA expression or ROS generation prevented NETosis in vitro and significantly reduced VTE in vivo. In conclusion, CC‐lEVs induce NETosis through the CYBA‐ROS‐citH3 pathway and increase VTE risk. Targeting CYBA expression or ROS production can provide novel strategies for preventing and treating VTE in high‐risk cancer patients.

## Graphical abstract

Venous thromboembolism (VTE) is a major threat to cancer patients. Cancer‐derived large extracellular vesicles deliver CYBA (p22phox) to neutrophils, triggering reactive oxygen species (ROS) and citrullinated histone H3 (citH3) formation. This drives neutrophil extracellular traps formation (NETosis), elevating VTE risk. Blocking CYBA or ROS prevented NETosis and reduced VTE, proposing new prevention strategies distinct from conventional anti‐coagulation.

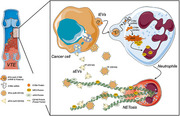

## 1 Introduction

Venous thromboembolism (VTE), encompassing deep vein thrombosis and pulmonary embolism, commonly emerges as an early complication in various cancers.^[^ ^1^, ^2^ ^]^ It affects approximate 15% to 20% of cancer patients, ranking as the second‐leading cause of death in this population while significantly impairing their quality of life.^[^ ^3^, ^4^ ^]^ The current management approaches for VTE in cancer patients face challenges due to the intricate interactions between anticoagulant therapy and cancer progression. These include a heightened bleeding risk from tumor invasion and chemotherapy‐induced thrombocytopenia.^[^ ^5^, ^6^ ^]^ Additionally, the effectiveness of treatments is diminished by the intrinsic thrombotic tendencies of cancer cells, which are further exacerbated by certain cancer therapies.^[^ ^7^ ^]^ Consequently, comprehensive research into the mechanisms of VTE in cancer is crucial to develop targeted strategies that improve patient outcomes and quality of life.

Neutrophil extracellular traps (NETs) are networks of extracellular fibers, primarily composed of DNA from neutrophils that bind various proteins, including histones and enzymes such as myeloperoxidase (MPO) and elastase.^[^ ^8^, ^9^ ^]^ NETs are involved in the pathogenesis of VTE by promoting coagulation, activating platelets, damaging endothelial cells, and inhibiting fibrinolysis.^[^ ^10^ ^]^ The process of NETs formation (NETosis) and the accompanied elevated biomarkers in VTE patients indicated their pivotal role in enhancing thrombotic processes.^[^ ^11^, ^12^ ^]^ Disrupting the formation or promoting the degradation of NETosis has been demonstrated to significantly reduce thrombus formation in many studies.^[^ ^13^, ^14^ ^]^ The intricate interplay between NETosis and VTE suggests an important intersection of cancer and coagulation, highlighting the potential of NETosis as a target for therapeutic intervention.

Extracellular vesicles (EVs), as crucial mediators of the tumor microenvironment, have been confirmed to be associated with NETosis. Su et al. have elucidated that lymphatic endothelial cells secrete CXCL8/2 in response to tumor‐derived EVs, inducing NETosis and promotion of lymph node metastasis,^[^ ^15^ ^]^ indicating that the complex communication between tumors and immune cells is associated with NETosis. Concurrently, the NETosis provides an attachment platform for tumor‐derived EVs, assisting their carried tissue factor (TF) to induce coagulation reactions and promote VTE.^[^ ^16^, ^17^, ^18^, ^19^ ^]^ However, the direct effects of tumor‐derived EVs induced NETosis and VTE remain unclear.

In this study, we demonstrated that cancer cell‐derived EVs (CC‐EVs), including small EVs (sEVs) and large EVs (lEVs), significantly induce NETosis. Further investigation revealed the activation of the cytochrome B‐245 light chain, reactive oxygen species, and citrullinated histone H3 signaling axis (CYBA‐ROS‐citH3) as a potential mechanism by which CC‐lEVs induce NETosis. As a subunit of NADPH oxidase complex (NOX2), CYBA is essential for ROS production. Elevated ROS levels trigger histone citrullination, leading to chromatin decondensation and NETs formation. This axis underscores the mechanistic link between CYBA delivery, oxidative stress, and NETosis. Additionally, in vivo experiments further confirmed that CC‐lEVs promote VTE through NETosis, and inhibiting CYBA expression or ROS production effectively blocked this pathological process, which may provide novel strategies for the clinical prevention and treatment of cancer patients at high risk for VTE.

## 2 Results

### 2.1 CC‐EVs Induced the NETosis

**Figure 1:**
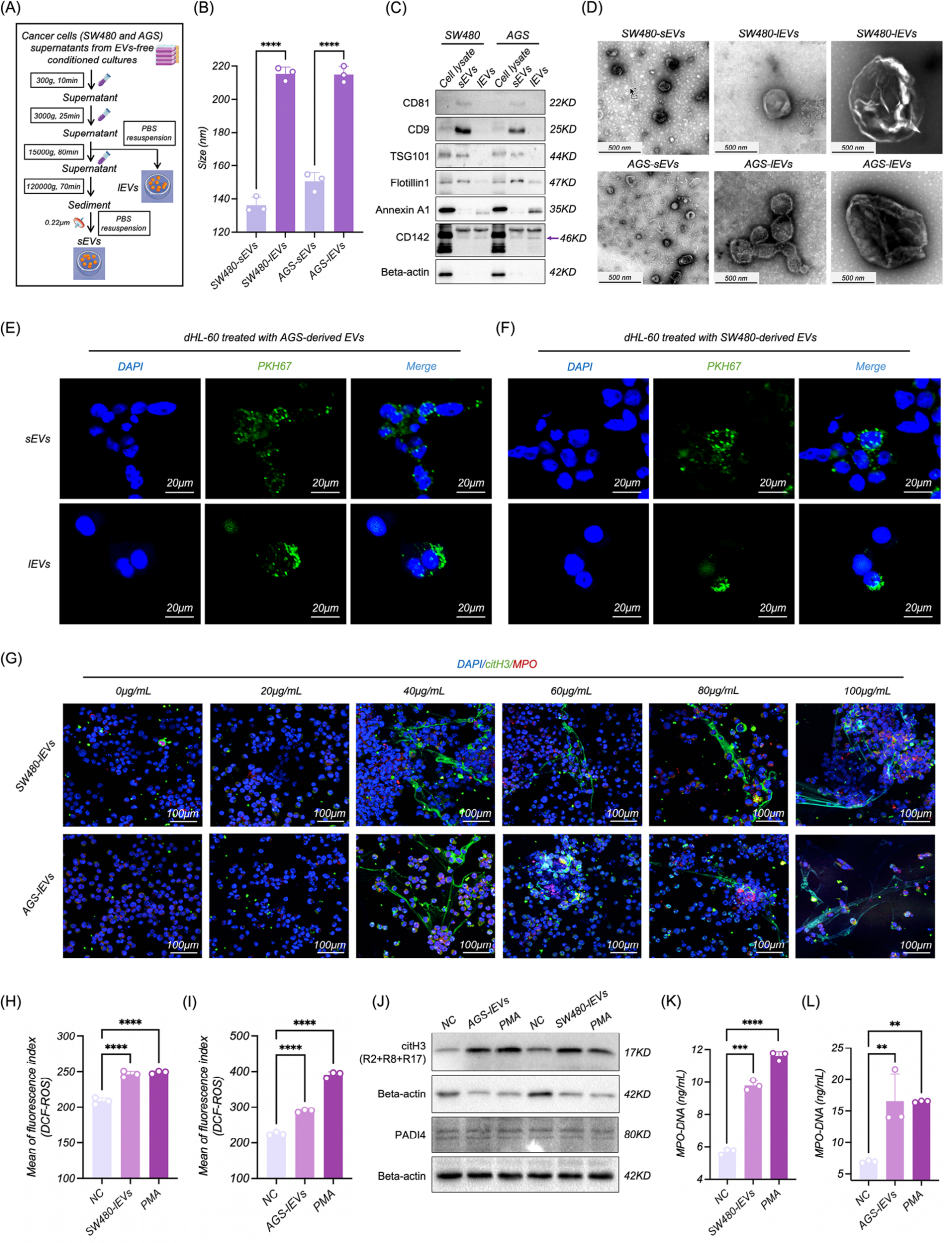
Isolation and characterization of cancer cell‐derived EVs and their role in the inducation of NETosis. A) Workflow for EVs isolation. B–D) Characterization of sEVs and lEVs by NTA (B), WB (C), and TEM (D). E,F) Confocal microscopy assessment of dHL‐60 uptake of Cancer cell‐derived sEVs and lEVs (CC‐sEVs and CC‐lEVs). G) Induction of NETosis in dHL‐60 by CC‐lEVs at different concentrations. H,I) Fluorescence analysis of ROS level in dHL‐60 treated with CC‐lEVs using a microplate reader (*n* = 3). J) WB analysis of citH3 and PAD4 expression in dHL‐60 treated with CC‐lEVs. K,L) ELISA quantification of MPO‐DNA complexes in the supernatants of dHL‐60 treated with CC‐lEVs (*n* = 3). All experiments were independently repeated three times, statistical results are presented as mean ± SD. Statistical significance was defined as ^*^ *P* < 0.05, ^**^ *P* < 0.01, ^***^ *P* < 0.001, and ^****^ *P* < 0.0001. NTA, Nanoparticle tracking analysis, WB, Western blot, TEM, Transmission electron microscope, ROS, Reactive oxygen species, citH3, citrullinated histone H3, PAD4, Peptidylarginine deiminase 4, ELISA, Enzyme‐linked immunosorbent assay, MPO‐DNA, Myeloperoxidase‐DNA complexes.

Given that gastric cancer and colorectal cancer are the main types of malignancies associated with high VTE risk,^[^ ^20^, ^21^, ^22^ ^]^ we cultured and collected the supernatants of AGS and SW480 cells to extract EVs. Based on an optimized workflow combining gradient ultracentrifugation (UC) with polyethersulfone (PES) filtration (**Figure** **1**A), we successfully isolated EVs with different sizes from AGS and SW480 cell culture supernatant. The separated lEVs and sEVs were confirmed to exhibit significant differences in size, enriched biomarkers, and morphology through nanoparticle tracking analysis (NTA), western blot (WB), and transmission electron microscope (TEM) (Figure 1B–D). Interestingly, we also observed that TF (CD142) was primarily carried by both lEVs and sEVs (Figure 1C), indicating that the main role of tumor‐derived EVs reported previously in promoting VTE via TF is attributed to both lEVs and sEVs.^[^ ^18^, ^19^ ^]^

As previously described,^[^ ^23^ ^]^ we induced HL‐60 into neutrophil‐like cells (dHL‐60) using 10 µM all‐trans retinoic acid (ATRA) to simulate the effects of EVs on neutrophils. Giemsa staining, WB and flow cytometry indicated that after 5 days of treatment with ARTA, HL‐60 exhibited nuclear lobulation and a significant upregulation of CD11b expression (Figure S1A–C, Supporting Information), suggesting successful induction of HL‐60 into dHL‐60. Then we confirmed dHL‐60 could effectively uptake CC‐EVs (Figure 1E,F), providing a research foundation for our subsequent investigations into their effects on NETosis.

To investigate the impact of CC‐EVs on neutrophils, we treated dHL‐60 seeded on poly‐L‐lysine‐coated coverslips with EVs (including lEVs and sEVs) at different concentrations. After 48 h, immunofluorescence staining (IF) showed that both lEVs and sEVs could induce NETosis when their concentrations exceeded 40 ug mL^−1^ (Figure 1G; Figure S1D, Supporting Information). Although sEVs exhibit a capacity to induce NETosis, their lower yield, diminished membrane protein enrichment, and weaker mechanistic relevance compared to lEVs underscore lEVs as the primary drivers of this process. Specifically, lEVs were isolated at a fourfold higher yield than sEVs from the same medium (Figure S2A,B, Supporting Information), which demonstrated that lEVs may be enriched in pro‐thrombotic and pro‐inflammatory cargo, including integrins, TF, and molecular mediators of platelet activation and aggregation, compared to the more selectively packaged sEVs.^[^ ^24^ ^]^ Mechanistically, lEVs are uniquely suited to deliver membrane‐associated proteins critical for ROS generation and NETosis. Key NADPH oxidase components are localized to the plasma membrane or endoplasmic reticulum.^[^ ^25^ ^]^ As lEVs are directly derived from plasma membrane budding, they inherently carry a higher abundance of membrane proteins compared to sEVs, which originate from endosomal pathways.^[^ ^26^ ^]^ This biogenetic distinction underscores the biological relevance of lEVs in mediating NETosis. Furthermore, lEV isolation via differential centrifugation offers technical advantages, including higher yield, reduced variability, and greater reproducibility compared to sEVs, which require UC or specialized kits prone to contamination. These attributes ensured experimental consistency, particularly for in vivo VTE models requiring large quantities of EVs. Consequently, subsequent studies focused on elucidating the role of lEV‐induced NETosis in VTE pathogenesis.

We used 90 nm Phorbol 12‐myristate 13‐acetate (PMA) as a positive inducer to further investigate NETosis‐associated biomarkers (Figure S3, Supporting Information).^[^ ^27^ ^]^ During the process of lEVs inducing NETosis, there was a significant increase in intracellular ROS levels and citH3 expression (Figure 1H–J), as well as concentration of myeloperoxidase‐DNA complexes (MPO‐DNA) in the cell supernatant (Figure 1K,L). The changes in these indicators validate the classical pathway of inducing NETosis,^[^ ^28^, ^29^ ^]^ wherein increased intracellular ROS and upregulation of citH3 expression led to the NETosis. It is important to note that while peptidylarginine deiminase 4 (PAD4) is considered a primary regulatory factor for citH3 expression,^[^ ^30^ ^]^ lEVs do not appear to influence the PAD4 level in dHL‐60 (Figure 1J). This suggested that the alteration of citH3 was contributed by other pathways.

### 2.2 CC‐lEVs‐Induced NETosis Depended on the Molecules They Carried

**Figure 2:**
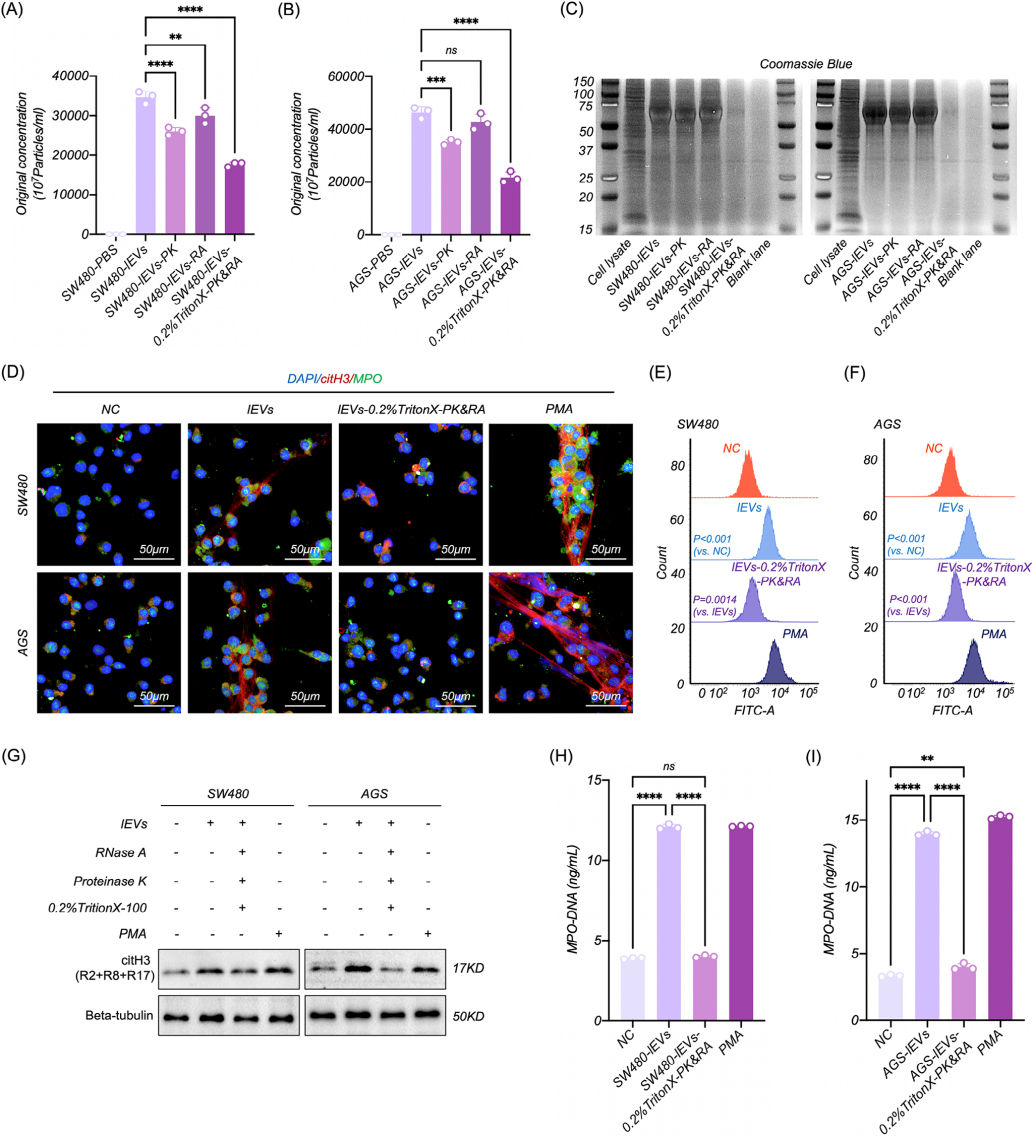
CC‐lEVs‐induced NETosis depended on the molecules they carried. A,B) NTA assessment of concentration of CC‐lEVs treated with Protease, RNase, and 0.2% TritonX‐100 (*n* = 3). C) Coomassie blue staining to evaluate protein degradation in CC‐lEVs treated with Protease, RNase, and 0.2% TritonX‐100. D) Confocal microscopy imaging of NETosis in dHL‐60 following treatment with RNP‐depleted CC‐lEVs. E,F) Flow cytometric analysis of ROS level in dHL‐60 treated with RNP‐depleted CC‐lEVs (*n* = 3). G) WB analysis of citH3 expression in dHL‐60 following treatment with RNP‐depleted CC‐lEVs. H,I) ELISA quantification of MPO‐DNA complexes in the supernatants of dHL‐60 treated with RNP‐depleted CC‐lEVs (*n* = 3). All experiments were independently repeated three times, statistical results are presented as mean ± SD. Statistical significance was defined as ^*^ *P* < 0.05, ^**^ *P* < 0.01, ^***^ *P* < 0.001, and ^****^ *P* < 0.0001. RNP, RNA, and protein, ROS, Reactive oxygen species, WB, Western blot, citH3, citrullinated histone H3, ELISA, Enzyme‐linked immunosorbent assay, MPO‐DNA, Myeloperoxidase‐DNA complexes.

To clarify whether the induction of NETosis by CC‐lEVs depends on the molecules they carried, lEVs were treated with Protease or RNase individually, or 0.2% TritonX‐100 in combination to deplete the RNA and protein of lEVs (RNP‐depleted EVs). NTA and Coomassie blue staining revealed that only the combined use of all three agents ensured complete degradation of the molecules by compromising the membrane structure of lEVs (**Figure** **2**A–C), consistent with previous reports.^[^ ^31^ ^]^ Compared to the lEVs and PMA treatment groups, we found that NETosis was absent (Figure 2D) and intracellular ROS level (Figure 2E,F), citH3 expression (Figure 2G), and MPO‐DNA of cell supernatant (Figure 2H,I) were reduced in dHL‐60 cells treated with RNP‐depleted lEVs. These results consistently indicated that CC‐lEV‐induced NETosis depended on the molecules they carried.

### 2.3 Bulk and Single‐Cell Transcriptomics Reveal CYBA May be a Key Effector Molecule in NETosis‐Mediated VTE

**Figure 3:**
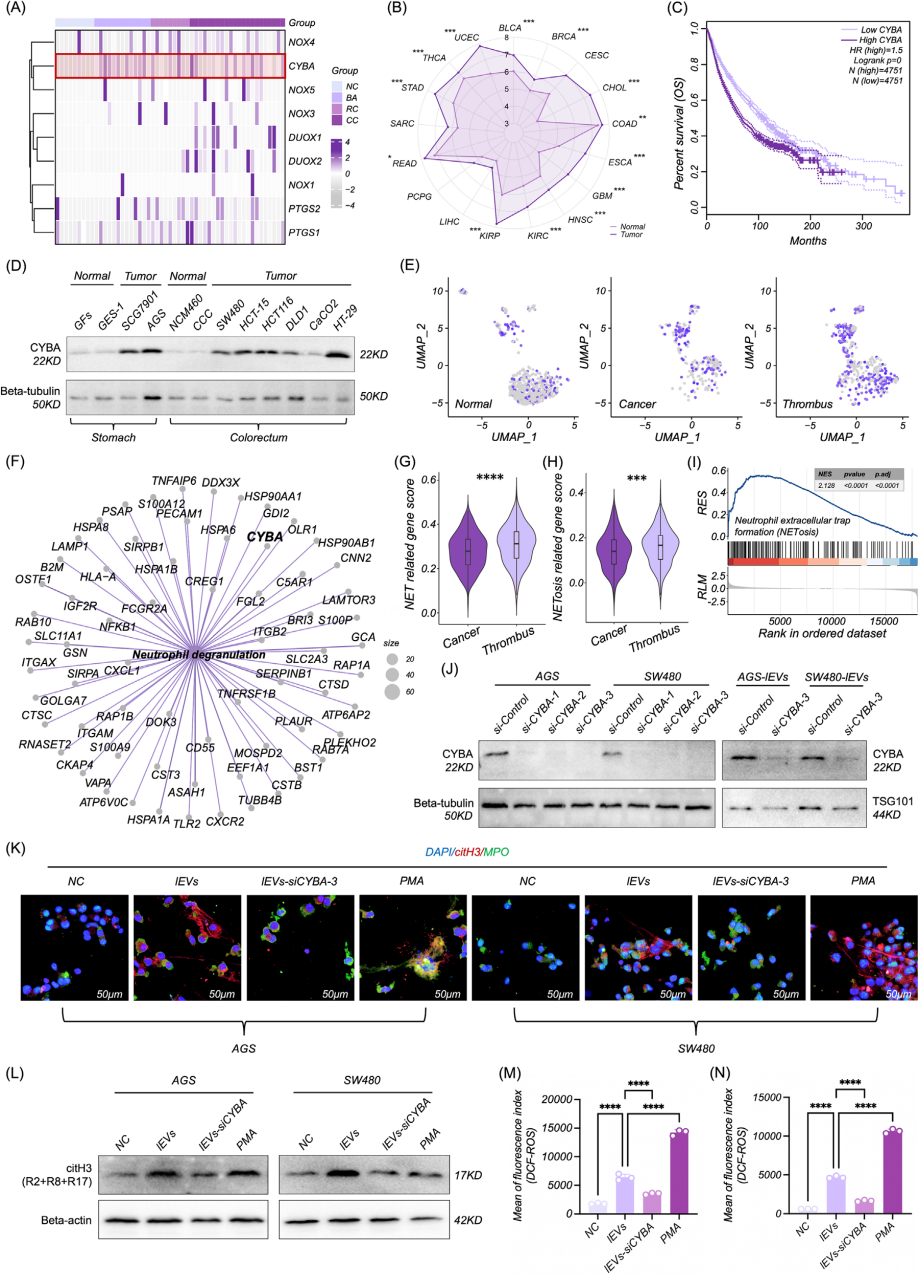
CYBA plays a key role in NETosis induced by CC‐lEVs. A) Heatmap illustrating differentially expressed genes related to oxidative stress in plasma EVs from tumor (22 colon cancer, 9 rectum cancer, and 13 benign adenoma) and nontumorous (*n* = 9) patients. B) Expression of *CYBA* in pan‐cancers (TCGA database). C) Prognostic significance of *CYBA* in pan‐cancers (The follow‐up duration for survival analysis was based on the clinical data available in the TCGA database, which includes patient survival information ranging from 0 to over 10 years, depending on the cancer type. The detailed information on the Cox Proportional Hazards Model can be found at the Http:14992768//gepia2.cancer‐pku.cn/#index. D) Expression of CYBA in cancer and non‐cancerous cell lines of the stomach and colon. E) Expression patterns of *CYBA* in normal, cancer, and thrombosis patient groups. F) The reactome enrichment analysis of differentially expressed genes in the thrombosis/cancer cohort (*CYBA* was enriched in NETosis‐associated pathways). G,H) Scoring of thrombosis and cancer cohort was performed based on literature‐curated genes (G) and the NETosis pathway gene set from KEGG database (H). I) GSEA analysis of NETosis pathway in the thrombosis compared to the tumor cohorts. J) Validation of CYBA knockdown in tumor cells and their derived lEVs. K) Confocal microscopy imaging of NETosis in dHL‐60 following treatment with CYBA‐knockdown CC‐lEVs. L) WB analysis of citH3 expression in dHL‐60 following treatment with CYBA‐knockdown CC‐lEVs. M,N) Flow cytometric analysis of ROS level in dHL‐60 treated with CYBA‐knockdown CC‐lEVs (*n* = 3). All experiments were independently repeated three times, statistical results are presented as mean ± SD. Statistical significance was defined as ^*^ *P* < 0.05, ^**^ *P* < 0.01, ^***^ *P* < 0.001, and ^****^ *P* < 0.0001. CYBA, Cytochrome B‐245 light chain, TCGA, The cancer genome atlas, RLM, Ranked list metric. RES, Running enrichment score. ROS, Reactive oxygen species.

Given that CC‐lEVs induce NETosis alongside a significant increase in intracellular ROS, the bioactive constituent within CC‐lEVs that facilitates NETosis induction may potentially be implicated in the modulation of ROS‐mediated signaling pathways. Therefore, we analyzed the RNA‐seq data of plasma EVs from nontumorous outpatients (n = 9) and gastrointestinal tumor patients (22 colon cancer, 9 rectum cancer, and 13 benign adenoma) to screen for differentially expressed genes related to oxidative stress (PRJNA639943, NCBI database). We found that among all ROS‐accosiated genes, the *CYBA* (logFC = 3.88, *P*_adj_ < 0.003) in plasma EVs of tumor patients was significantly higher than nontumorous controls (**Figure** **3**A; Table S1, Supporting Information). In pan‐cancer (TCGA database, the normalization method used for gene expression analysis was FPKM), including tumors with a high incidence of VTE such as glioblastoma (GBM), stomach adenocarcinoma (STAD), colon adenocarcinoma (COAD), rectal adenocarcinoma (READ), and kidney renal clear cell carcinoma (KIRC),^[^ ^32^ ^]^ *CYBA* was also significantly increased and associated with worse overall survival (Figure 3B,C). Consistently, CYBA expression levels were higher in gastric and colorectal cancer cell lines compared to non‐cancerous cell lines (Figure 3D). A significant increase in intracellular CYBA levels was observed in dHL‐60 after co‐culture with CC‐lEVs (Figure S4, Supporting Information). Hence, CYBA could be a possible effector molecule that contributes to CC‐lEVs‐induced NETosis.

Furthermore, we used single‐cell RNA sequencing (scRNA‐seq) to investigate the regulatory role of *CYBA* in NETosis within cancer‐associated thrombotic niches. ScRNA‐seq of KIRC thrombi identified eight myeloid cell subpopulations, including a distinct neutrophil cluster characterized by elevated expression of canonical markers (*S100A8*, *FCGR3B*, and *CXCL8*, Figure S5A, Supporting Information). Differential gene expression analysis revealed pronounced upregulation of *CYBA* in thrombus‐associated neutrophils compared to those in primary tumors and normal controls (Figure 3E; Figure S5B, Supporting Information). Functional enrichment analyses further linked thrombus neutrophils to pathways critical for NETosis, including “neutrophil chemotaxis”, “neutrophil degranulation”, and “ROS‐related processes,”^[^ ^33^, ^34^, ^35^ ^]^ with *CYBA* emerging as a central regulator across these pathways (Figure 3F; Figure S5C,D, Supporting Information).

Neutrophil functional scoring using NET‐associated gene sets (Tables S2 and S3, Supporting Information) confirmed enhanced NET‐forming activity in thrombus microenvironments (Figure 3G,H). Bulk RNA sequencing of paired thrombus and primary tumor tissues (PRJNA596338, N = 30, NCBI database) validated these findings, with gene set enrichment analysis (GSEA) demonstrating significant activation of NETosis pathway in thrombi (Figure 3I). *CYBA* was robustly enriched within this pathway, aligning with its role in ROS‐mediated NETosis. These multi‐omics approaches establish *CYBA* as a key molecular driver of NETosis in cancer‐associated thrombosis. The convergence of single‐cell heterogeneity and bulk transcriptomic signatures underscores the spatial and functional dominance of *CYBA* in thrombus microenvironments, providing mechanistic insights into its role in bridging neutrophil activation to thromboinflammatory cascades.

### 2.4 CYBA was Responsible for the NETosis Induced by CC‐lEVs

To investigate the possible role of CYBA in CC‐lEV‐induced NETosis, we knocked down CYBA in cancer cells and their derived lEVs (Figure 3J). Treatment of dHL‐60 with CYBA‐knockdown lEVs failed to induce NETosis (Figure 3K), and no notable increase in citH3 level (Figure 3L) or intracellular ROS was observed (Figure 3M,N). We further overexpressed CYBA in cancer cells and their derived lEVs (Figure S6A–C, Supporting Information). Although NETosis was observed in both dHL‐60 treated with control lEVs and CYBA‐overexpressed lEVs (Figure S7A, Supporting Information), there was no significant increase in intracellular ROS and citH3 level between control lEV‐ and CYBA‐overexpressed lEV‐treated groups (Figure S7B–D, Supporting Information). Taken together, CYBA plays a key role in triggering NETosis but may not be a major contributing factor to the exacerbation of NETosis progression.

### 2.5 Inhibition of CC‐lEVs Uptake Suppressed the NETosis

The uptake of CC‐lEVs by neutrophils constitutes the initial step in NETosis, and inhibition of this cellular uptake process may represent a potential therapeutic strategy for NETosis prevention. Thus, we co‐cultured dHL‐60 with lEVs‐PKH67 and three different inhibitors targeting distinct pathways of EVs uptake (including endocytosis, caveolae‐dependent endocytosis, and clathrin‐dependent endocytosis).

**Figure 4:**
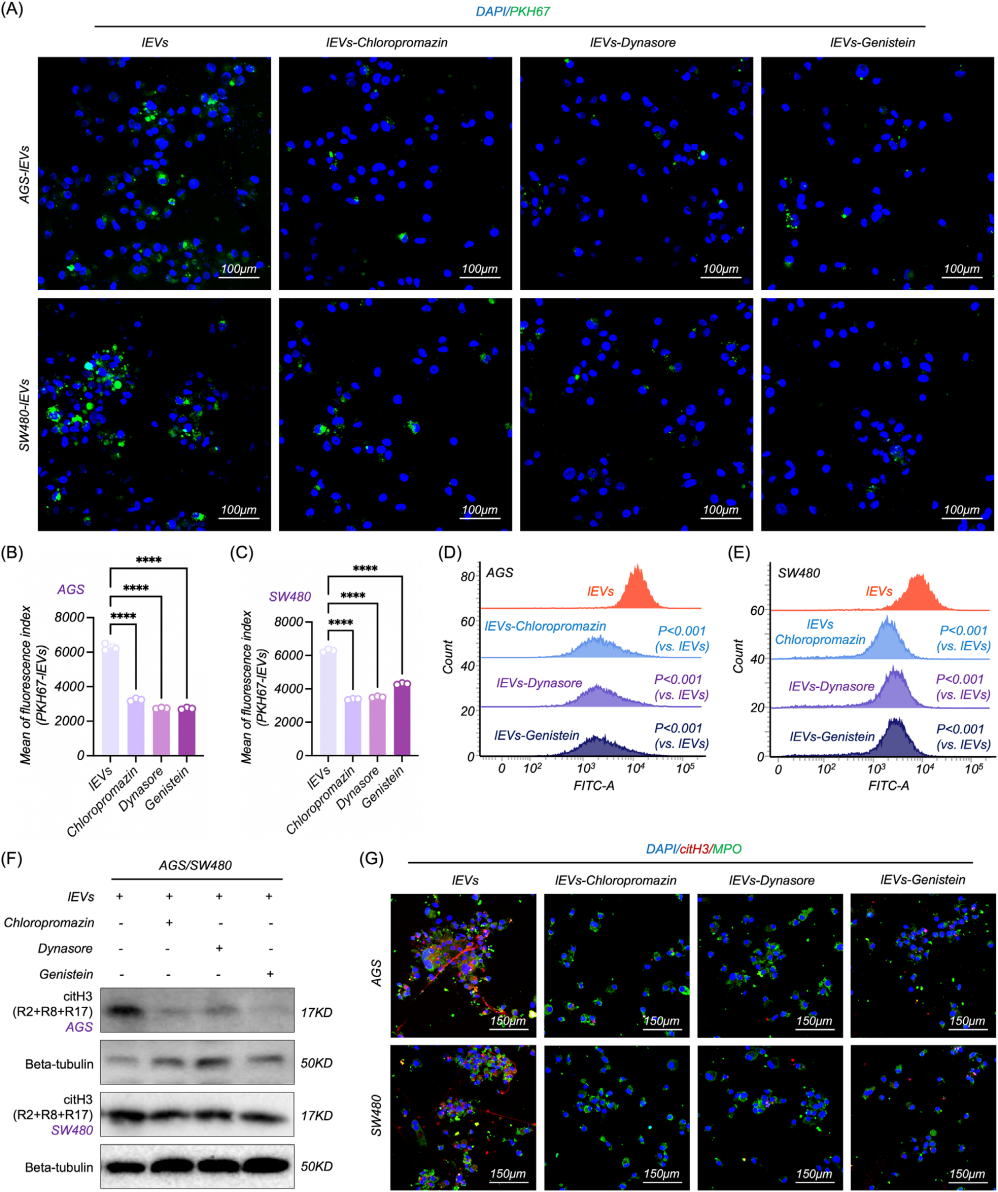
Inhibition of CC‐lEVs uptake suppressed the NETosis. A) Confocal microscopy imaging of lEVs uptake in dHL‐60 following treatment with different inhibitors targeting distinct pathways of EVs uptake, including endocytosis (Dynasore), caveolae‐dependent endocytosis (Genistein), and clathrin‐dependent endocytosis (Chloropromazin). B,C) Flow cytometric analysis of CC‐lEVs uptake efficiency in dHL‐60 treated with Chloropromazine, Dynasore, and Genistein (*n* = 3). D,E). Flow cytometric analysis of ROS level in dHL‐60 treated with CC‐lEVs and different inhibitors targeting distinct pathways of EVs uptake (*n* = 3). F) WB analysis of citH3 expression in dHL‐60 following treatment with CC‐lEVs and different inhibitors targeting distinct pathways of EVs uptake. G) Confocal microscopy imaging of NETosis in dHL‐60 following treatment with CC‐lEVs and different inhibitors targeting distinct pathways of EVs uptake. All experiments were independently repeated three times, statistical results are presented as mean ± SD. Statistical significance was defined as ^*^ *P* < 0.05, ^**^ *P* < 0.01, ^***^ *P* < 0.001, and ^****^ *P* < 0.0001. ROS, Reactive oxygen species, WB, Western blot.

After 12 h, we found that the uptake efficiency of lEVs was obviously decreased in all inhibitor‐treated groups (**Figure** **4**A–C). Additionally, intracellular ROS and citH3 levels were significantly reduced (Figure 4D–F), and no NETosis was observed in these groups (Figure 4G). These results suggested that inhibition of CC‐lEVs uptake successfully suppressed NETosis.

### 2.6 CC‐lEVs Induced the NETosis via CYBA‐ROS‐citH3 Axis

**Figure 5:**
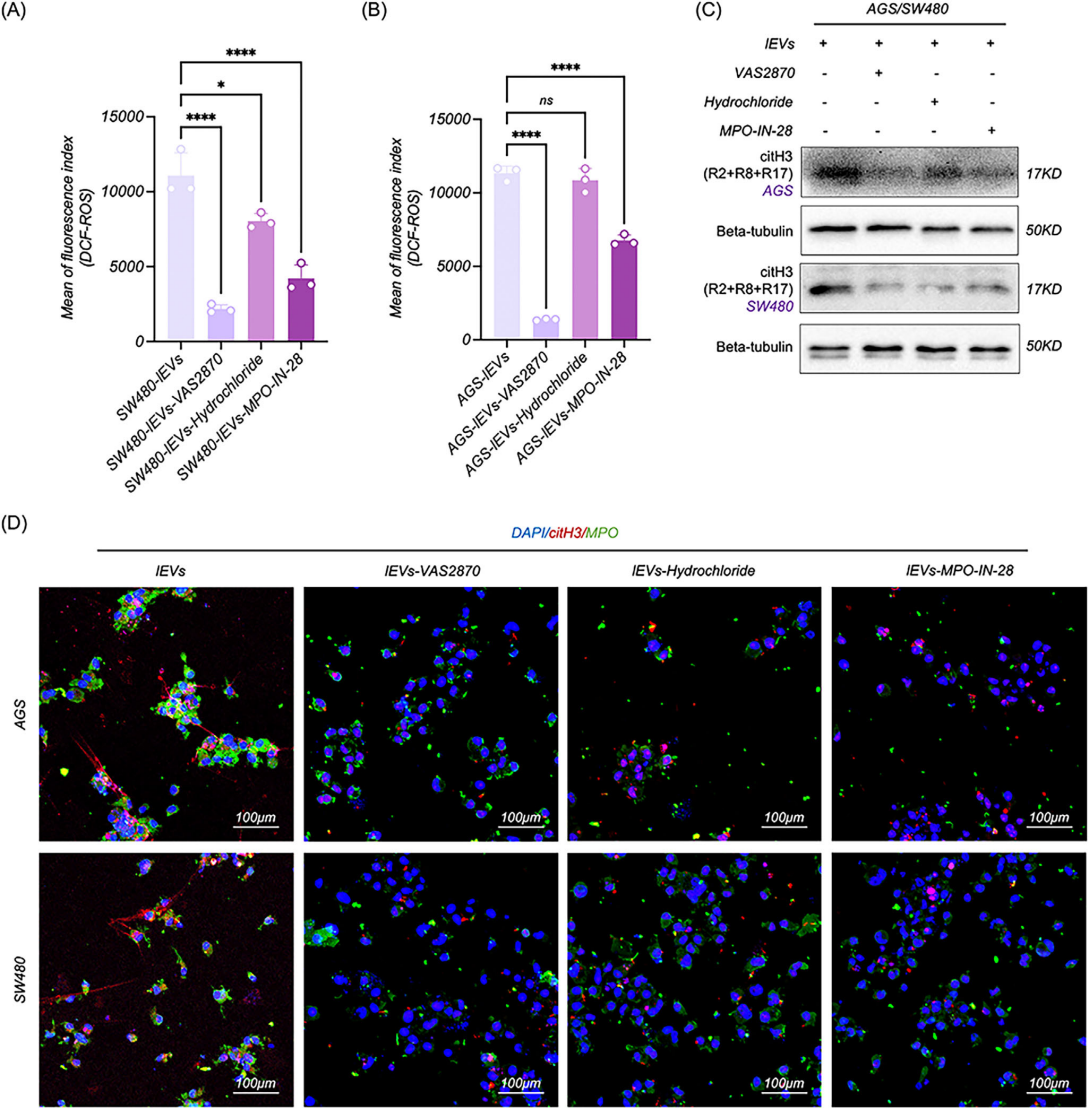
CC‐lEVs induced the NETosis via CYBA‐ROS‐citH3 axis. A,B) Flow cytometric analysis of ROS level in dHL‐60 treated with CC‐lEVs and different inhibitors targeting ROS, PAD4, and MPO (*n* = 3). C) WB analysis of citH3 expression in dHL‐60 following treatment with CC‐lEVs and different inhibitors targeting ROS, PAD4, and MPO. D) Confocal microscopy imaging of NETosis in dHL‐60 following treatment with CC‐lEVs and different inhibitors targeting ROS, PAD4, and MPO. All experiments were independently repeated three times, statistical results are presented as mean ± SD. Statistical significance was defined as ^*^ *P* < 0.05, ^**^ *P* < 0.01, ^***^ *P* < 0.001, and ^****^ *P* < 0.0001. CYBA, Cytochrome B‐245 light chain, ROS, Reactive oxygen species, citH3, citrullinated histone H3, WB, Western blot, PAD4, Peptidylarginine deiminase 4.

Previously, we demonstrated the significant role of lEV‐derived CYBA in inducing NETosis, which significantly impacted intracellular ROS levels and citH3 expression. To further elucidate the role of ROS and citH3 in CC‐lEV‐induced NETosis, we added VAS2870 (ROS inhibitor), MPO‐IN‐28 (MPO inhibitor), and Hydrochloride (PAD4 inhibitor) into CC‐lEV‐treated dHL‐60 cells. As expected, we observed that VAS2870 and MPO‐IN‐28 significantly reduced the ROS levels of dHL‐60 (**Figure** **5**A,B), which led to the downregulation and absence of citH3 and NETosis (Figure 5C,D). Although Hydrochloride showed no significant inhibitory effects on ROS, it could hinder NETosis of dHL‐60 by suppressing citH3 expression (Figure 5A–D). The above results indicated that CC‐lEV‐induced NETosis through the CYBA‐ROS‐citH3 axis, but this pathological phenomenon could be rescued by inhibiting ROS levels and citH3 expression.

### 2.7 CC‐lEVs Promoted VTE by Inducing the NETosis

**Figure 6:**
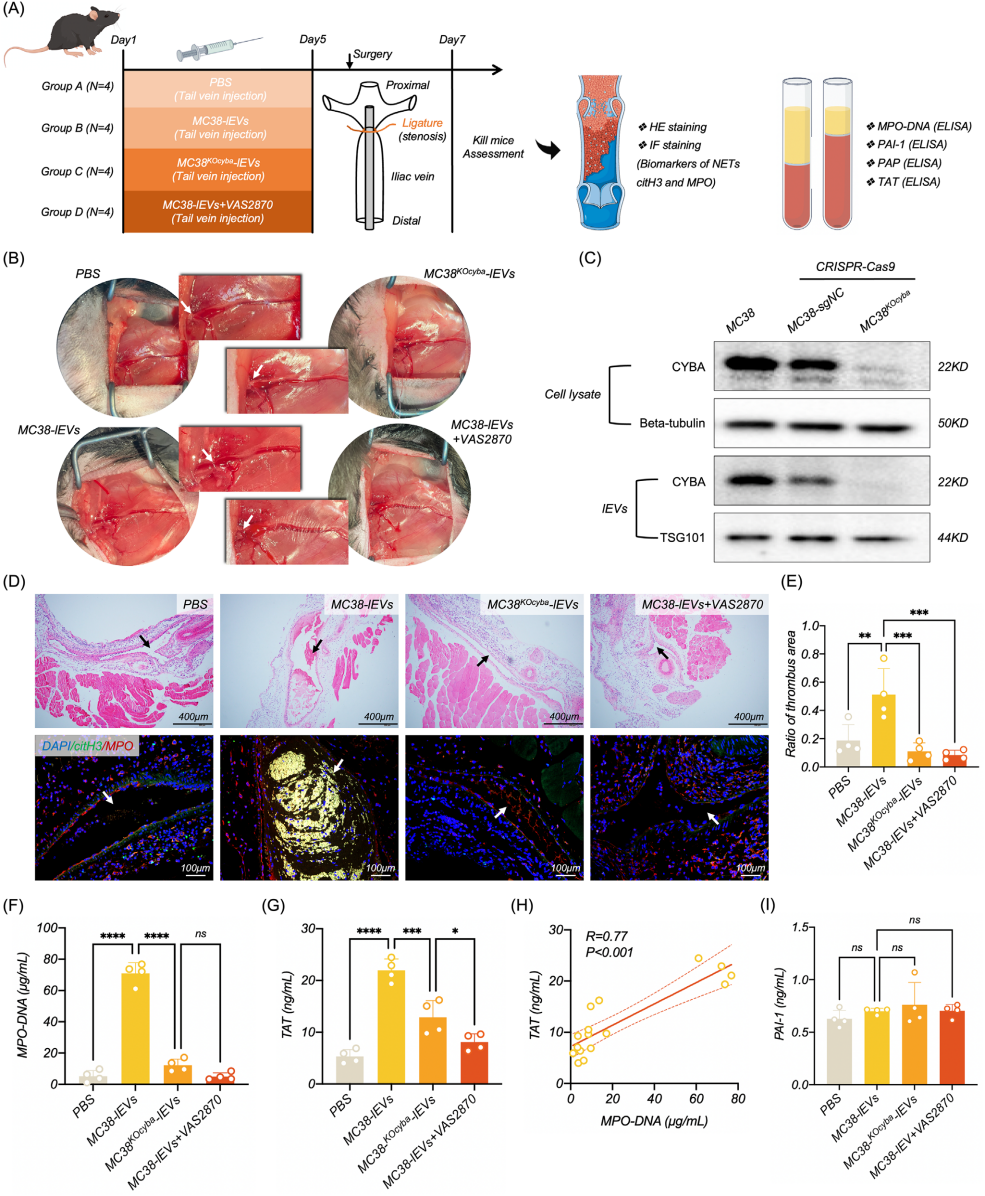
CC‐lEVs promoted VTE by inducing the NETosis. A) Workflow and evaluation indicators for establishing the VTE mouse model (*n* = 4). B) Photographic representation of the VTE mouse model, with white arrows indicating the constriction site on the left iliac vein (*n* = 4). (C) WB validation of *cyba* knockout efficiency in MC38 using CRISPR‐Cas9. D) H&E staining and confocal microscopy to assess the formation of thrombus and NETosis in the iliac vein across different treatment groups (*n* = 4). E) Quantification of thrombus area based on H&E staining among various treatment groups (*n* = 4). F,G) ELISA measurements of plasma MPO‐DNA and TAT complexes in different treatment groups (*n* = 4). H) Correlation analysis between plasma MPO‐DNA and TAT complexes (*n* = 4). (I) ELISA measurement of plasma PAI‐1 level among different treatment groups (*n* = 4). Statistical results are presented as mean ± SD. Statistical significance was defined as ^*^ *P* < 0.05, ^**^ *P* < 0.01, ^***^ *P* < 0.001, and ^****^ *P* < 0.0001. VTE, Venous thromboembolism, WB, Western blot, H&E, Hematoxylin‐eosin, ELISA, Enzyme‐linked immunosorbent assay, MPO‐DNA, Myeloperoxidase‐DNA complexes, TAT, Thrombin‐antithrombin complex, PAI‐1, Plasminogen activator inhibitor‐1.

NETosis is closely associated with increased VTE risk.^[^ ^36^ ^]^ To clarify whether CC‐lEVs enhanced the VTE risk by inducing NETosis, we designed an in vivo experiment for validation. Mice were divided into four groups as illustrated in **Figure** **6**A: 1) the Control group, which received PBS injections, 2) the lEVs group, which received CC‐lEVs, 3) the *cyba*‐KO lEVs group, which received lEVs derived from MC38 with *cyba* knockdown, and 4) the NETosis inhibitor group, which received CC‐lEVs along with a ROS inhibitor (VAS8970). PBS, CC‐lEVs, and VAS2870 were continuously injected into C57BL mice via the tail vein for 5 days. Six hours after completing the injections on the 5th day, an iliac vein constriction surgery was performed (Figure 6B), followed by collection of plasma and thrombus tissues 48 h post‐surgery.

In this in vivo experiment, we utilized the CRISPR/Cas9 system to knock out *cyba* in MC38 cells and collected their lEVs (MC38^KO^ *^cyba^*‐lEVs) (Figure 6C). Hematoxylin‐eosin (H&E) staining revealed more pronounced acute VTE in mice treated with MC38‐lEVs, and IF staining also showed significantly higher levels of NETosis in these VTEs. Knockout of *cyba* or use of VAS2870 markedly inhibited VTE and NETosis (Figure 6D,E). Furthermore, Enzyme linked immunosorbent assays (ELISA) were used to detect NETosis‐ and VTE‐related biomarkers in plasma and the results showed MPO‐DNA and thrombin‐antithrombin complex (TAT) levels were highest in the MC38‐lEVs treatment group (Figure 6F,G), with a significant positive correlation between the two biomarkers (Figure 6H). Knockout of *cyba* or use of VAS2870 similarly led to an obvious reduction in MPO‐DNA and TAT levels (Figure 6F,G). However, there were no significant differences in plasminogen activator inhibitor‐1 (PAI‐1) levels among these groups (Figure 6I). Overall, the in vivo experiment revealed that CC‐lEVs could promote VTE by inducing NETosis.

## 3 Discussion

Cancer patients frequently exhibit a hypercoagulable state, which arises from a combination of factors, such as the upregulation of procoagulant factors (e.g., TF) and systemic inflammatory responses.^[^ ^37^, ^38^ ^]^ These factors contribute to the development of VTE and significantly increase the mortality risk associated with thrombosis‐related complications.^[^ ^39^, ^40^ ^]^ Although a series of anticoagulant prophylactic measures and thrombolytic interventions targeting these factors have made progress and improved the prognosis for some patients,^[^ ^41^, ^42^ ^]^ limited understanding of the intricate mechanisms underlying thrombus occurrence and progression has resulted it being challenging to achieve more definitive treatment.

From the perspective of intercellular communication, we revealed that CC‐lEVs increased the VTE risk of cancer patients by inducing NETosis. lEVs delivered CYBA from cancer cells to neutrophils, triggering elevated ROS and upregulated citH3 expression in target cells, which resulted in the release of decondensed chromatin (DNA and histones) and formed a trapping network. As a crucial component of the NOX2, CYBA participates in the regulation of intracellular ROS levels (Figure S8, Supporting Information) and immune responses.^[^ ^43^, ^44^ ^]^ Upregulated CYBA was observed in various malignancies (Figure 3B), facilitating cancer cells to sustain proliferation and invasiveness via ROS‐associated signaling pathways.^[^ ^45^ ^]^ Therefore, it is reasonable and explicable that aberrant CYBA within neutrophils induces NETosis by regulating ROS. The generation of ROS is essential for initiating NETosis. It not only directly induced chromatin decondensation by reducing the activity of methyltransferases but also activated the NF‐kB and MAPK signaling pathways to regulate citH3 expression, thereby indirectly inducing NETosis.^[^ ^46^, ^47^, ^48^ ^]^ Consequently, the formation of NETs does not necessarily rely on the completion of the classical pathway mediated by PAD4. In our study, no significant alterations of PAD4 expression were observed in dHL‐60 treated with CC‐lEVs, suggesting that the upregulation of citH3 is primarily mediated through ROS‐activated signaling pathways. Additionally, emerging evidence of MPO‐mediated oxidative stress and PAD isoform compensation may explain this phenomenon: First, the MPO‐rich microenvironment of neutrophils enables ROS‐mediated oxidative stress to synergize with chromatin modification processes. MPO‐generated reactive species may potentiate post‐translational activation of existing PAD4 or other isoforms, bypassing the need for de novo PAD4 synthesis.^[^ ^49^ ^]^ Second, compensatory mechanisms through other PAD family members could maintain citrullination capacity, as PAD2 has demonstrated partial functional redundancy with PAD4 in certain inflammatory contexts.^[^ ^50^ ^]^

It is worth noting that we found that CC‐lEVs naturally carrying CYBA (at concentrations≥40 µg mL^−1^) were sufficient to induce robust NETosis in dHL‐60, marked by elevated ROS, citH3, and NETs. However, overexpression of CYBA in lEVs did not further amplify these effects. This suggests that endogenous CYBA levels in lEVs already saturate the molecular threshold required to activate the NOX2, which generates ROS—a critical driver of NETosis. Beyond this threshold, additional CYBA may not enhance ROS production or downstream NETosis, consistent with studies showing that NOX2 activity is tightly regulated by assembly/activation dynamics rather than CYBA abundance alone.^[^ ^51^ ^]^ Additionally, while CYBA is essential for NOX2 assembly, other subunits (e.g., gp91phox, p47phox, and p67phox) or signaling pathways (e.g., PKC, ERK) may become rate‐limiting in overexpressing conditions.^[^ ^52^, ^53^ ^]^ Thus, even with excess CYBA, the lack of proportional increases in ROS or citH3 implies that subsequent steps in NETosis signaling are constrained. This finding is consistent with our observation that lEVs with CYBA knockdown abolished NETosis, confirming the indispensable role of CYBA as an initiator. Finally, the absence of exacerbation by CYBA overexpression highlights a biologically optimized mechanism in cancer: CYBA delivery ensures baseline NETosis to promote VTE, but further amplification may be counterproductive or physiologically unnecessary. This aligns with the concept that excessive NETosis could lead to unintended cytotoxicity or immune dysregulation, which cancers might evolutionarily avoid through compensatory mechanisms.^[^ ^54^, ^55^ ^]^ Such mechanisms may include the upregulation of antioxidant systems or the activation of negative regulators of NADPH oxidase. These adaptive strategies likely serve to maintain a balance that supports tumor progression while avoiding detrimental systemic consequences.

The DNA scaffold released by NETosis serves as a platform to attract and activate coagulation factors, facilitating platelet aggregation and thrombin generation, thereby accelerating thrombus formation.^[^ ^56^ ^]^ Our in vivo experiments further demonstrated a strong correlation between MPO‐DNA and thrombin levels, with increased NETosis deposition observed in larger thrombi. These findings collectively underscore that elevated NETosis significantly enhances the risk of VTE. Additionally, as a crucial fibrinolysis inhibitor, PAI‐1 exerts its effects by binding to and suppressing the activity of tissue plasminogen activator and urokinase plasminogen activator, thereby impeding the activation of plasminogen and influencing the balance between coagulation and fibrinolysis.^[^ ^57^ ^]^ Previous studies have also reported that the DNA and protein components of NETs could modulate the PAI‐1 expression by interacting with cell membrane receptors such as receptor of advanced glycation endproducts and toll‐like receptor.^[^ ^58^, ^59^, ^60^ ^]^ However, our investigation did not unveil a definitive association between NETosis and PAI‐1. This discrepancy may be attributed to potential negative regulatory factors, such as miRNAs (Figures S9 and S10, Supporting Information), present in CC‐lEVs, which could impede the PAI‐1 expression.^[^ ^61^, ^62^, ^63^ ^]^

**Figure 7:**
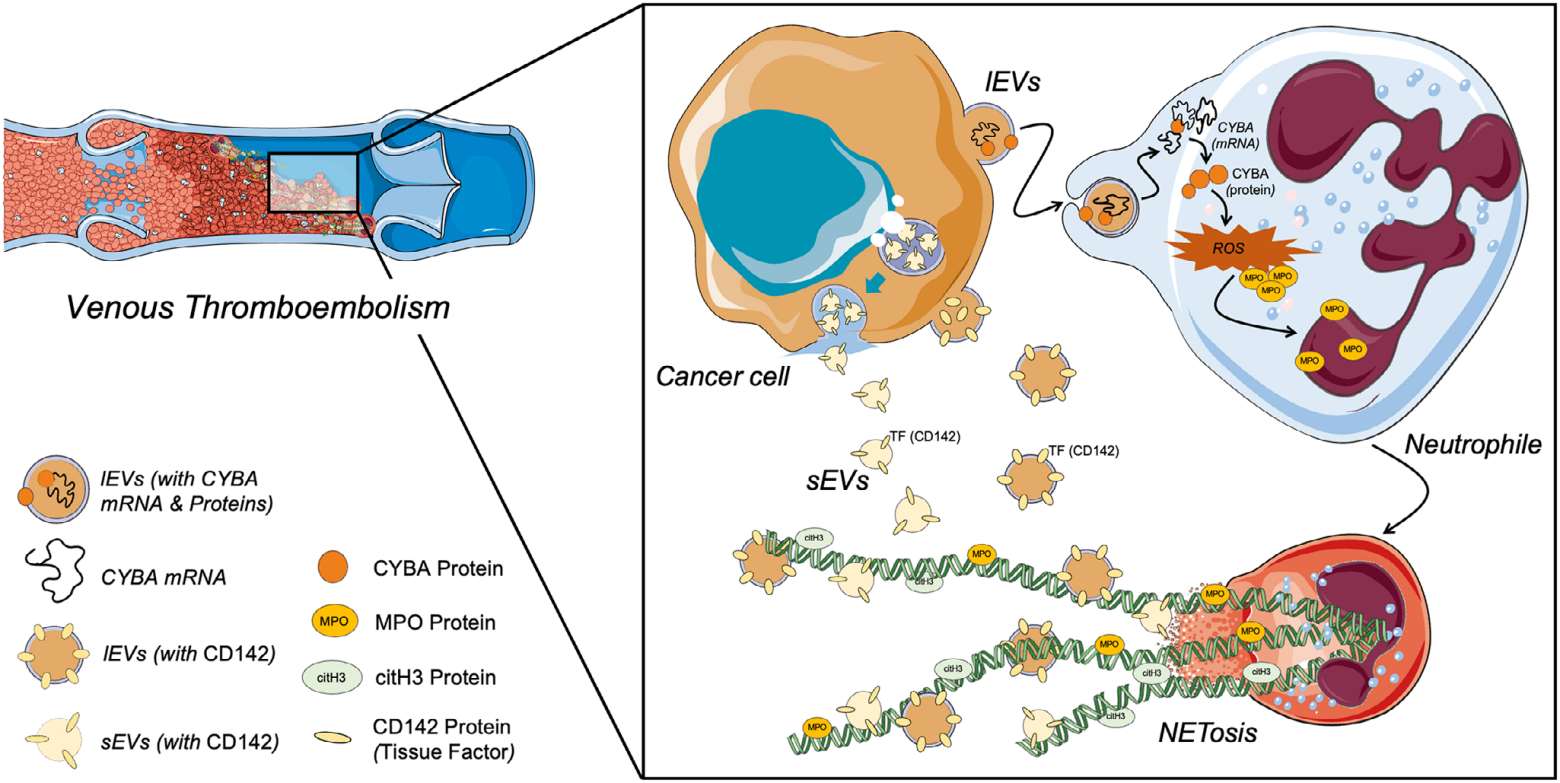
Proposed molecular mechanism by which CC‐lEVs promote venous thromboembolism via NETosis.

The targeted deletion of PAD4 markedly attenuates the NETosis and the prevalence of related diseases.^[^ ^64^ ^]^ The absence of PAD4 hampered the enzymatic process of histone arginine deamination, crucial for chromatin decondensation, thereby disrupting the necessary nucleosome relaxation for NETosis.^[^ ^65^ ^]^ Our study revealed that a reduction in intracellular ROS levels constitutes an additional, potent strategy to inhibit NETosis. Elevated ROS induced both DNA base damage and single‐strand breaks that promoted DNA unraveling, while simultaneously activating neutrophils to undergo apoptosis processes that together drove NETosis.^[^ ^66^, ^67^ ^]^ Moreover, rescue experiments have shown that ROS inhibition correlated with decreased citH3 levels, further substantiating the role of ROS in modulating citH3 expression and influencing NETosis dynamics. Additionally, during the characterization of biomarkers in EVs, we observed that TF was expressed in both sEVs and lEVs, with a marginally higher expression level in lEVs (Figure 1C). As a critical initiator of the coagulation cascade, TF activates the extrinsic pathway of coagulation and amplifies the coagulation process through interactions with other coagulation factors.^[^ ^68^, ^69^ ^]^ Crucially, existing evidence reveals a self‐reinforcing interplay between TF and NETosis: TF‐enriched NETs contribute to thrombogenesis (e.g., myocardial infarction),^[^ ^70^ ^]^ while NETs‐derived histones and granular proteins conversely upregulate endothelial TF expression, creating a prothrombotic feedforward cycle.^[^ ^71^ ^]^ This bidirectional crosstalk mechanistically explains our findings that CC‐lEVs effectively promote venous thrombosis formation through direct activation of the coagulation cascade and indirect facilitation of NETosis (**Figure** **7**). In future studies, we will evaluate the synergistic effects of NETosis with other prothrombotic factors. For instance, pro‐inflammatory cytokines such as IL‐8 and TNF‐α are known to enhance neutrophil activation and NETosis while simultaneously promoting endothelial dysfunction and coagulation.^[^ ^72^, ^73^ ^]^ It is plausible that lEVs, in conjunction with these cytokines, could establish a prothrombotic microenvironment that exacerbates NETosis and VTE formation. Further investigation into these interactions may provide deeper insights into the mechanisms underlying VTE pathogenesis and identify potential therapeutic targets.

Our study contains several limitations requiring critical analysis. A primary constraint stems from incomplete mechanistic understanding of tumor‐derived sEVs in NETosis‐mediated VTE pathogenesis. While we identified correlations between sEV exposure and NETosis, the precise molecular cascades through which sEVs initiate or amplify neutrophil extracellular trap formation remain undefined (e.g., CYBA‐ROS‐citH3 axis). This limitation is particularly consequential given the fundamental biological divergence between sEVs and lEVs. Resolving this requires systematic investigations combining EV subtype‐selective isolation techniques with neutrophil‐specific receptor knockout models to disambiguate sEV‐specific contributions from lEV‐mediated effects.^[^ ^74^ ^]^ Concurrent spatiotemporal mapping of sEV thrombogenic cargos (e.g., TF‐positive subsets) within evolving thrombi could further elucidate their microenvironmental regulation of NETosis. Such mechanistic clarification would advance both biological understanding and therapeutic targeting of EV subpopulations in cancer‐associated thrombosis.

Another limitation concerns the generalizability of our findings derived from gastric and colorectal cancer models. While TCGA pan‐cancer analysis reveals widespread *CYBA* overexpression across malignancies with high VTE risk, suggesting potential universality of lEV‐mediated NETosis induction. However, the functional validation herein remains confined to two cancer types. The generalizability of the CYBA‐lEVs mechanism requires further experimental confirmation across a broader range of solid tumors. Additionally, Critical tumor‐specific variables, including intertumoral heterogeneity in EV cargo composition and tumor microenvironmental factors influencing NETosis modulation, remain uncharacterized and warrant systematic exploration. Future studies should establish whether CYBA operates as a central regulator or functions within broader molecular networks across malignancies with distinct thrombotic risk profiles. Comprehensive EV characterization paired with clinical correlation analyses will be essential to delineate context‐dependent contributions of tumor‐derived EVs to VTE pathogenesis.

## 4 Clinical Relevance

The inhibition of CYBA/ROS signaling represents a promising therapeutic strategy, particularly for cancer‐associated VTE. The CYBA‐containing NADPH oxidase complexes play a pivotal role in driving excessive ROS production, which is crucial for NETosis induction and subsequent VTE formation. Targeting this pathway could offer significant clinical benefits by mitigating NETosis‐driven thrombotic events in cancer patients, who are at a substantially elevated risk of VTE. By inhibiting CYBA/ROS, it may be possible to disrupt the pathological cascade linking CC‐lEVs, NETosis, and VTE, thereby providing a novel therapeutic approach to reduce thrombotic complications in oncology. Several NADPH oxidase inhibitors have been investigated for their potential to block ROS production and could be repurposed for this context. For instance, apocynin, a well‐studied NADPH oxidase inhibitor, prevents the assembly of the NOX2 complex, thereby reducing ROS generation and showing efficacy in preclinical models of inflammation and thrombosis.^[^ ^75^ ^]^ Similarly, diphenyleneiodonium, a potent inhibitor of flavoproteins including NADPH oxidases, has demonstrated the ability to suppress ROS production and NETosis in experimental settings.^[^ ^76^ ^]^ Additionally, GKT137831 (Setanaxib), a selective NOX1/4 inhibitor currently under clinical investigation for fibrotic diseases, could also be explored for its potential to inhibit NETosis and VTE in cancer patients.^[^ ^77^ ^]^ However, translational considerations must address pharmacological limitations, such as apocynin requires metabolic activation for efficacy and exhibits off‐target interactions with MPO;^[^ ^78^ ^]^ DPI non‐selectively inhibits mitochondrial complexes, risking cellular toxicity.^[^ ^79^ ^]^ While these inhibitors show promise, further preclinical and clinical studies are essential to evaluate their efficacy and safety specifically in the context of cancer‐associated VTE.

Systemic inhibition of CYBA or ROS may disrupt essential physiological functions, necessitating a more targeted therapeutic approach. Future strategies could focus on selectively targeting CC‐lEVs or their specific interactions with immune cells, such as neutrophil, rather than broadly inhibiting CYBA or ROS systemically. This approach could minimize off‐target effects while maintaining therapeutic efficacy. The timing and context of CYBA inhibition are also critical, transient or localized inhibition during high‐risk periods for VTE formation, such as post‐surgery or during chemotherapy,^[^ ^80^, ^81^ ^]^ might reduce the risk of adverse effects while still preventing NETosis and VTE. Furthermore, combining CYBA‐targeted strategies with other therapeutic approaches, such as anticoagulants or anti‐inflammatory agents, could enhance efficacy while allowing for lower doses of CYBA inhibitors, thereby reducing potential side effects. This multifaceted approach could provide a more balanced and effective treatment paradigm for managing cancer‐associated VTE.

## 5 Conclusion

In this study, we establish for the first time that CC‐lEVs, alongside CC‐sEVs, significantly augment the risk of VTE in cancer patients. Our findings reveal a novel mechanism by which CC‐lEVs facilitate VTE through the CYBA‐ROS‐citH3 pathways, which is distinct from the conventional TF‐mediated coagulation pathway. We demonstrate that inhibiting CYBA expression or ROS production effectively blocks this pathological process, highlighting innovative preventive strategies that target CC‐EVs to mitigate the incidence of cancer‐associated VTE. These insights provide valuable guidance for the clinical prevention and treatment of cancer patients at high risk for VTE.

## 6 Experimental Section

### Ethics Statement

The mice were raised in a pathogen‐free environment at the Laboratory Animals Center of Beijing Friendship Hospital. They were humanely euthanized through carbon dioxide narcosis in compliance with both local and national ethical guidelines of laboratory animal use. The Institutional Animal Care and Use Committee at the Beijing Friendship Hospital granted approval for all animal procedures and experimental protocols.

### RNA‐Seq Data Analysis of Plasmas Derived EVs of Tumor Patients

RNA‐seq data of plasmas derived EVs of tumor patients, including 22 colon cancer (CC) patients, 9 rectum cancer (RC) patients, 13 benign adenoma (BA), and 9 non‐cancerous outpatients (NC), were obtained from our previous report.^[^ ^82^ ^]^ The RNA‐seq data have been deposited at the sequence read archive database of NCBI (PRJNA639943).

Differential expression of mRNAs was conducted using DESeq2, a widely used R package for differential expression analysis of count data derived from high‐throughput sequencing experiments. DESeq2 employs a negative binomial distribution model to account for overdispersion in read counts and applies shrinkage estimation for fold changes to improve the stability and accuracy of results. The analysis included normalization of raw read counts using the median of ratios method, followed by hypothesis testing using the Wald test to assess statistical significance. Genes with an adjusted *P* (*P*_adj_) value < 0.05 and |log2FC|>1.5 were considered differentially expressed. Heatmap was conducted using ggplot2 package.

### ScRNA‐Seq and Bulk RNA‐Seq of Caval Venous Tumor Thrombi in Cancer Patients

The ScRNA‐seq data were obtained from the National Genomics Data Center at the Beijing Institute of Genomics, Chinese Academy of Sciences (HRA000963), encompassing caval venous tumor thrombi and matched specimens from clear cell renal cell carcinoma patients. Myeloid cell subclustering was performed using Seurat (v4.4.0) with unsupervised clustering (resolution = 0.6), yielding 14 initial clusters that were annotated based on canonical marker genes. Neutrophils were identified via a combinatorial signature (*S100A8, IFITM2, FCGR3B, CXCL8, G0S2*), culminating in the systematic classification of eight myeloid subsets. Differential gene expression analysis between thrombus and cancer groups within neutrophil subpopulations was conducted using the FindMarkers function (Wilcoxon rank‐sum test) with stringent thresholds (absolute log2FC ≥ 0.25, expression in≥10% of cells, Bonferroni‐corrected p‐values). Significantly dysregulated genes underwent functional enrichment analysis via Gene Ontology biological processes and Reactome pathways. NETosis activity was quantified using AddModuleScore, which computes functional scores based on a curated NET‐associated gene set.

Bulk RNA‐seq data were acquired from the NCBI Sequence Read Archive (PRJNA596338). Initial quality assessment was performed using FastQC (v0.12.1), followed by adapter trimming and low‐quality base removal with Trimmomatic (v0.39). High‐quality reads were aligned to the human reference genome GRCh38 (GENCODE p14) using HISAT2 (v2.2.1) with default parameters. Processed BAM files were sorted, indexed, and converted using Samtools (v1.21), and gene‐level counts were quantified via featureCounts (Subread v1.5.3) with paired‐end correction (‐p) and default settings. For differential expression analysis between thrombus and cancer groups, DESeq2 (v1.38.3) was employed after filtering genes undetected in > 25% of samples. Differentially expressed genes were defined by *P*_adj_ < 0.05 (Benjamini‐Hochberg) and absolute log2FC≥1.2. GSEA was conducted using clusterProfiler (v4.6.2) with KEGG pathways, where significance was determined by adjusted *P* < 0.05 and pathway activation directionality inferred from normalized enrichment scores (NES>0: activation, NES < 0: suppression).

### Animal Study

C57BL/6 mice were purchased from the Beijing Vital River Laboratory Animal Technology Co., Ltd. through the Model Animals Research Center of Beijing Friendship Hospital. Considering the well‐documented association between estrogen and increased VTE,^[^ ^83^, ^84^ ^]^ male mice aged 9–10 weeks were selected for this study. The mice were randomized in different groups (N = 4 mice/group), PBS (0.1 mL kg^−1^), MC38‐lEVs (1.6 × 10^11^ particle/kg), MC38‐KO*_cyba_*‐lEVs (1.6 × 10^11^ particle/kg), and MC38‐lEVs plus ROS inhibitor (VAS2870, HY‐12804, MCE, USA) (1.6×10^11^ particle/kg plus 2.5 mg kg^−1^). All mice were injected lEVs suspension or ROS inhibitor via the tail vein. They were housed in a controlled environment with regulated light and temperature and were provided ad libitum access to food and water.

### Venous Thrombosis Model

Left common iliac vein ligation (LCIVL) was performed based on the previously reported inferior vena cava ligation procedure.^[^ ^85^, ^86^, ^87^ ^]^ Briefly, mice were anesthetized with isoflurane (R510‐22‐10, RWD, China) and placed in a supine position. The LCIVL was exposed by blunt dissection and was ligated by an 8.0 polypropylene suture immediately at the proximal end over an unclogging needle (outer diameter = 0.15 mm). The needle was placed outside the vessel so that piercing or any other injury to the LCIVL wall was completely avoided. After being ligated, the needle was removed to obtain a partial flow restriction. Muscle, peritoneum, and skin were closed by monofilament absorbable suture and 6.0 silk, respectively. Mice were killed after 48 h, and thrombi developed in the LCIVL were taken for analysis.

Blood was collected into 7.6% sodium citrate (1:20 v/v) from anesthetized mice. The citrated blood was centrifuged at 3600 rpm for 15 min at 4 °C to obtain plasma, which was immediately stored at −80 °C until further ELISA.

### Cell Lines and Cell Culture

SW480, AGS, and MC38 cells were obtained from the Cell bank of the Chinese Academy of Sciences and were cultured with RPMI 1640 (03.4007C, Eallbio, China), F‐12/DMEM (SH30023.01, cytiva, USA), and DMEM (MA0564, Meilunbio, China) mediums contained 10% EVs‐free FBS (EXO‐FBS‐50A‐1, Biosharp, China). HL‐60 (CL‐0110, Pricella, China) and THP‐1 (CL‐0233, Pricella, China) cells were cultured with PRMI 1640 medium contained 20% FBS (10100147, Thermo Fisher, USA) and 1% Penicillin‐Streptomycin (P1400, Solarbio, China). All cell lines were authenticated for STR analysis, exhibiting a negative result for mycoplasma contamination. THP‐1 cells were treated with 100 ng/mL phorbol 12‐myristate 13‐acetate (PMA, 79346, Sigma–Aldrich, USA) for 24 h for differentiation into M0 macrophages, then 100 ng mL^−1^ lipopolysaccharides (LPS, L8880, Solarbio, China) plus 20 ng mL^−1^ interferon gamma (IFN‐γ, P00028, Solarbio, China) were further used to stimulate M0 into M1 macrophages after 48 h.^[^ ^88^, ^89^ ^]^

### Cell Transfection

pcDNA3.1‐*CYBA*‐Flag was synthesized by Youbio Co., Ltd (Changsha, China). siRNA (si*CYBA*‐1, si*CYBA*‐2, and si*CYBA*‐3) was designed based on Invitrogen Block‐iT RNAi Designer (https://rnaidesigner.thermofisher.com/rnaiexpress/) and purchased from GenePharma (Suzhou, China) (Table S4, Supporting Information). The SW480 and AGS cells were seeded in T225 culture flask (721003, NEST, China). When the cell had reached 70% confluence, we transfected the plasmid and siRNA according to the manufacturer's instructions using MegaTran 2.0 (TT210003, Origene, USA) and siTran 2.0 (TT320002, Origene, USA). Cells and supernatant were collected at 72 h after transfection for further experiments. The transfection efficiency (Overexpression and knockdown) was validated via western blotting.

### EVs Isolation and Quantification

Conditioned medium of SW480, AGS, and MC38 was gathered after culturing with 10% EVs‐free FBS for 72 h. Cells and cell debris were first removed by centrifugation (300 g for 10 min and 3000 g for 25 min), then the supernatant was centrifuged at 15 000 g for 80 min, and the sediments (lEVs) were resuspended in PBS. Finally, the supernatant was further centrifuged at 120 000 g for 70 min to isolate sEVs. sEVs were further filtered through 0.22 µm PES membranes. All centrifugation procedures were proceeded under 4 °C using Hitachi (CF16RXI2, Hitachi, Japan) and Beckman ultracentrifuges (Optima XE‐100, Beckman, USA). Pierce BCA Protein Assay Kit (23225, Thermo Fisher, USA) was used for quantifying the protein concentration at an absorbance of 562 nm. EVs isolated were stored at ‐80 °C before use.

### EVs Labeling and Characterization

The particle concentration and size distribution of EVs were measured by nanoparticle analysis (PMX 110, ZetaView, Germany). Western blot (*Western blotting of Methods section*) was used for characterization of EVs biomarkers. After staining with 1% (v/v) urayl acetate dihydrate (YS25690U, YaJi, China), EVs microstructure was observed by a transmission electron microscope (TEM, HT7800, Hitachi, Japan).

### Differentiation of Neutrophil‐Like HL‐60 and Wright‐Giemsa Staining

To differentiate HL‐60 cells into neutrophil‐like cells, HL‐60 was treated with 10 µm ATRA  (R2625, Sigma–Aldrich, USA) for 5 days as described previously.^[^ ^90^, ^91^ ^]^ Wright‐Giemsa stain solution (G1020, Solarbio, China) was used to visualize the lobed nucleus of dHL‐60. Images were captured using an inverted microscope (DP72, Olympus, Japan).

### CRISPR‐Cas9 Genome Editing

To achieve the deletion of *cyba*, a specific guide RNA (sgRNA) targeting *cyba* was designed (sgRNA1: 5′‐GGGGGCATCGTGGCTACTGCTGG‐3′). Oligonucleotides were synthesized and ligated into Lentivirus‐Cas9 (OBiO, China). MC38 cells were transfected with the vectors containing sgRNA sequence using the Lipofectamine 8000 (C0533FT, Beyotime, China) according to the manufacturer's protocol. Finally, clonal selection of the cells was performed, and knockout efficiency was validated by western blotting.

### Intracellular ROS Detection

Intracellular ROS (cyto‐ROS) was detected using the ROS‐sensitive dye DCFH‐DA (S0033M, Beyotime, China). After incubating the probe for 30 min, the concentration of cyto‐ROS was quantified by a microplate reader (Molecular Devices, SpectraMax M3) at 488/525 (Excitement/Emission) nm and FACS (Becton‐Dicknson, FACSCalibur) at 488 nm.

### Fluorescence Staining

Anti‐Rabbit/Mouse, Alexa fluor plus 488 (A11034/A32723, Invitrogen, USA) and anti‐Rabbit/Mouse, Alexa fluor plus 594 (A11012/A11005, Invitrogen, USA) were used to locate NETosis (MPO and citH3). Briefly, dHL‐60 seeded to poly lysine coated slides (YALy20‐PLL, ACMEC, China) or serial (5 µm) sections of paraffin‐embedded thrombus were fixed in 4% paraformaldehyde (PFA, BL539A, Biosharp, China) for 30 min and permeabilized with 0.3% Triton X‐100 for 20 min. slides or sections were blocked with 5% BSA (A8020, Solarbio, China) at room temperature (RT) and incubated with anti‐citH3 (1:1500, ab281584, Abcam, UK) and anti‐MPO (1:300, 66177‐1‐Ig, Proteintech, China) overnight at 4 °C. The following day, the slides or sections were incubated with fluorescently labeled secondary antibodies at RT for 2 h and mounted with 4′6‐diamidino‐2‐phenylindole (DAPI, sc‐3598, Santa Cruz, USA). For the EVs uptake assays, PKH67 (PKH67GL, Sigma‐Aldrich, USA) was used for the labeling of EVs. 200 µL EVs were mixed with 1 µL PKH67 reagent at RT, and 400 µL EVs‐free FBS was used to stop the staining. Images were captured using a confocal microscope (FV1200, Olympus, Japan).

### Histological Analyses

Mice thrombus samples were fixed in 4% PFA in overnight, paraffin‐embedded, and cut into 5 µm thick sections. To evaluate the morphology and composition of thrombus, H&E staining was conducted as previously described.^[^ ^92^, ^93^ ^]^ Tissue sections were deparaffinized in xylene and rehydrated through an alcohol series before staining with hematoxylin for 5–10 min. Subsequent differentiation in acid alcohol and counterstaining with eosin for 1–2 min were conducted, followed by dehydration, clearing in xylene, mounting with neutral balsam, and coverslipping. The stained slides were allowed to dry thoroughly before microscopic examination to assess thrombus morphology and architecture meticulously. The thrombus area was measured using Image J (1.53k).

### Enzyme‐Linked Immunosorbent Assay

Levels of MPO‐DNA (MM‐2467H1, Meimian, China, MM‐1177M1, Meimian, China), PAI‐1 (MM‐0066M2, Meimian, China), and TAT (MM‐0424M2, Meimian, China) were evaluated by ELISA Kits according to the manufacturer's instructions. The lowest detection limit was 0.1 ng/mL for human MPO‐DNA, 1.0 ug mL^−1^ for mouse MPO‐DNA, 1.0 ng mL^−1^ for PAI‐1, and 1.0 ng mL^−1^ for TAT. The assay plates were read at 450 nm.

### Western Blotting

As previously described,^[^ ^94^ ^]^ proteins were extracted from dHL‐60 cells using a RIPA lysis buffer (P0013C, Beyotime, China) containing protease (04693116001, Roche, China) and phosphatase (CW2383S, CWBIO, China) inhibitors. Lysed suspension was centrifuged at 12 000 rcf for 12 min at 4 °C. The supernatant was collected and quantified using the BCA assay kit (BL521A, Biosharp, China). Then the protein extracts were heated at 95 °C for 10 min, and an equivalent of 20 ug protein was separated by electrophoresis in 12.5% SDS‐PAGE (PG114, EpiZyme, China). The proteins were then transferred to PVDF membranes (IPVH00010, Millipore, USA), and the membranes were blocked with 8% non‐fat milk for 2 h at room temperature. After washing three times with TBST, the membranes were incubated with the appropriate primary antibodies overnight at 4 °C. After washing six times with TBST, the membranes were incubated with HRP‐conjugated secondary antibodies for 1 h at room temperature. Protein signals were detected with enhanced chemiluminescence reagent (1810202, CLiNX, China) and analyzed by molecular Imager (BIO‐RAD, ChemiDocXRS+). The following primary antibodies were used: CD81 (1:5000, ab109201, Abcam, UK), CD9 (1:20000, 60232‐1‐Ig, Proteintech, China), TSG101 (1:20000, 67381‐1‐Ig, Proteintech, China), Flotillin1 (1:5000, 15571‐1‐AP, Proteintech, China), Annexin A1 (1:20000, 66344‐1‐Ig, Proteintech, China), CD142 (1:1000, bs‐4690R, Bioss, China), CD11B (1:2000, 66519‐1‐Ig, Proteintech, China), CitH3 (1:1000, ab281584, Abcam, UK), PADI4 (1:3000, 17373‐1‐AP, Proteintech, China), CYBA (1:1000, bs‐3879R, Bioss, China, 1:1000, A10694, ABclonal, China), Flag (1:20 000, 66008‐4‐Ig, Proteintech, China), GAPDH (1:50000, 60004‐1‐Ig, Proteintech, China) β‐actin (1:50000, AC026, ABclonal, China), and β‐tubulin (1:6000, 10094‐1‐AP, Proteintech, China). Goat anti‐Rabbit (1:50000, E030120‐01, EarthOx, USA) and Goat anti‐Mouse (1:50000, E030110‐01, EarthOx, USA) were secondary antibodies used in this study.

### Statistical Analysis

All statistical analyses were performed using GraphPad Prism 9.0 (San Diego, CA, USA) and R version 4.2.3 (www.r‐project.org). The flow cytometry data were evaluated based on the mean fluorescence intensity. For comparisons between two groups, the Student's t‐test was applied, while one‐way analysis of variance (ANOVA) was utilized for comparisons involving three or more groups. Survival analysis between groups was conducted using the Kaplan‐Meier method, with statistical significance assessed by the log‐rank test. Data are expressed as mean ± SD. Statistical significance was defined as ^*^ *P* < 0.05, ^**^ *P* < 0.01, ^***^ *P* < 0.001, and ^****^ *P* < 0.0001.

### Ethics Approval

The animal studies were approved by the Animal Care and Use Committee of Beijing Friendship Hospital, China. All mice were fed under standard laboratory conditions free of specific pathogens.

## Conflict of Interest

The authors declare no conflict of interest.

## Author Contributions

L.M. and Y.X. contributed to the study design. X.L., Y.J., C.X., and S.M. conducted the experiments. Data analysis was carried out by X.L., L.S., Q.G., and M.L. The manuscript was written by X.L. and L.M. X.L. created Figures 1, 2, 3, 4, 5, 6 and L.M. created Figure 7. All authors reviewed and approved the final version of the manuscript.

## Supporting information



Supporting Information

Supporting Information

Supporting Information

Supporting Information

## Data Availability

The data that support the findings of this study are available in the supplementary material of this article.
