# Supplementary material for: Cancer Cell‐Derived Large Extracellular Vesicles Promote Venous Thromboembolism by Activating NETosis Through Delivering CYBA
Source: Adv Sci (Weinh). 2025 Jul 21;12(37):e07867. doi: 10.1002/advs.202507867 (PMC12499506; doi:10.1002/advs.202507867)
Supplement: Supplementary file 1 — Supporting Information [file ADVS-12-e07867-s002.docx]

**Cancer Cell-Derived Large Extracellular Vesicles Promote Venous Thromboembolism by Activating NETosis through delivering CYBA**

Xiangji Li^1^, Yingjiao Ju^2^, Chenjie Xu^1^, Shixiang Ma^3^, Lan Sun^4^, Qingdong Guo^1^,

Mingyuan Liu^5^, Yibin Xie^6*^, Li Min^1,2*^

^1^*Department of Gastroenterology, Beijing Friendship Hospital, Capital Medical University, State Key Laboratory of Digestie Health, National Clinical Research Center for Digestive Diseases, Beijing Digestive Disease Center, Beijing Key Laboratory for Precancerous Lesion of Digestive Disease, Beijing 100050, P. R. China*

*^2^Research Center, Beijing Clinical Research Institute, Beijing Friendship Hospital, Capital Medical University, Beijing 100050, P. R. China*

^3^*Department of Retroperitoneal Tumor Surgery, Peking University International Hospital, Beijing 102206, P. R. China.*

^4^*Institute of Basic Research in Clinical Medicine, China Academy of Chinese Medical Sciences, Beijing 100700, P. R. China.*

^5^*Department of Vascular Surgery, Beijing Friendship Hospital, Capital Medical University, Beijing 100050, P. R. China*

^6^*Department of Pancreatic and Gastric Surgery, National Cancer Center/National
Clinical Research Center for Cancer/Cancer Hospital, Chinese Academy of Medical
Sciences and Peking Union Medical College, Beijing 100021, P. R. China.*

****Correspondence should be addressed to:***

*Prof. Li Min: Department of Gastroenterology, Beijing Friendship Hospital, Capital Medical University, Beijing 100050, China. E-mail: [minli@ccmu.edu.cn](mailto:minli@ccmu.edu.cn).

**Prof. Yibin Xie: Department of Pancreatic and Gastric Surgery, National CancerCenter/National Clinical Research Center for Cancer/Cancer Hospital, Chinese Academy of Medical Sciences and Peking Union Medical College, Beijing 100021,

China. E-mail: [yibinxie@cicams.ac.cn](mailto:yibinxie@cicams.ac.cn).

**APPENDIX A. SUPPLEMENTARY DATA**

**CONTENTS**

**1. siRNA sequences used for *CYBA* knockdown** (*Table S4*)

**2. CC-sEVs induced the NETosis in dHL-60** (*Fig. S1*)

**3. Analysis of protein concentrations of sEVs and lEVs in conditioned media from homologous cells** (*Fig. S2*)

**4. Exploration of positive control (PMA) concentration** (*Fig. S3*)

**5. Validation of tumor-derived lEVs delivering CYBA to dHL-60** (*Fig. S4*)

**6. Single-cell transcriptomics reveal *CYBA* may be a key effector molecule in NETosis-mediated VTE** (*Fig. S5*)

**7. Validation of *CYBA* overexpression in AGS and SW480 and their lEVs** (*Fig. S6*)

**8. CYBA may not be a major contributing factor to exacerbation of NETosis induced by CC-lEVs** (*Fig. S7*)

**9. Analysis of *CYBA* biological function** (*Fig. S8*)

**10. MiRNAs negatively correlated with *PAI-1 (SERPINE1)* expression in TCGA-STAD** (*Fig. S9*)

**11. MiRNAs negatively correlated with *PAI-1 (SERPINE1)* expression in TCGA-COAD and TCGA-READ** (*Fig. S10*)

**12. Complete membrane and Uncropped bands of *Fig. 1C***

**13. Complete membrane and Uncropped bands of *Fig. 1J***

**14. Complete membrane and Uncropped bands of *Fig. 2G***

**15. Complete membrane and Uncropped bands of *Fig. 3D***

**16. Complete membrane and Uncropped bands of *Fig. 3J***

**17. Complete membrane and Uncropped bands of *Fig. 3L***

**18. Complete membrane and Uncropped bands of *Fig. 4F***

**19. Complete membrane and Uncropped bands of *Fig. 5C***

**20. Complete membrane and Uncropped bands of *Fig. 6C***

**21. Complete membrane and Uncropped bands of** *Fig. S1B*

**22. Complete membrane and Uncropped bands of** *Fig. S4*

**23. Complete membrane and Uncropped bands of** *Fig. S6*

**24. Complete membrane and Uncropped bands of** *Fig. S7D*

**1. Table S4. siRNA sequences used for *CYBA* knockdown.**

| siRNA | Sequence |
| --- | --- |
| si-*CYBA*#1 | F: 5’-CCAGGUGCACCCACCUGCAAUAAAU-3’ |
|  | R: 5’-AUUUAUUGCAGGUGGGUGCACCUGG-3’ |
| si-*CYBA*#2 | F: 5’-CAGGUGCACCCACCUGCAAUAAAUG-3’ |
| si-*CYBA*#3 | R: 5’-CAUUUAUUGCAGGUGGGUGCACCUG-3’  F: 5’-CCCACCUGCAAUAAAUGCAGCGAAG-3’  R: 5’-CUUCGCUGCAUUUAUUGCAGGUGGG-3’ |
| si-Ctrl | F: 5’-CCAGUACCCACCCGUACUAAGGAAU-3’ |
|  | R: 5’-AUUCCUUAGUACGGGUGGGUACUGG-3’ |

*Note: si-CYBA, CYBA small interfering RNA; si-Ctrl, negative control small interfering RNA.*

**2. Fig. S1. CC-sEVs induced the NETosis in dHL-60.** (A) Giemsa staining assessment of ATRA-induced differentiation of HL-60 into dHL-60. (B-C) WB and flow cytometry analysis of the expression of the CD11B (granulocytic biomarker) in dHL-60. (D) Induction of NETosis in dHL-60 by CC-sEVs at different concentrations.

***
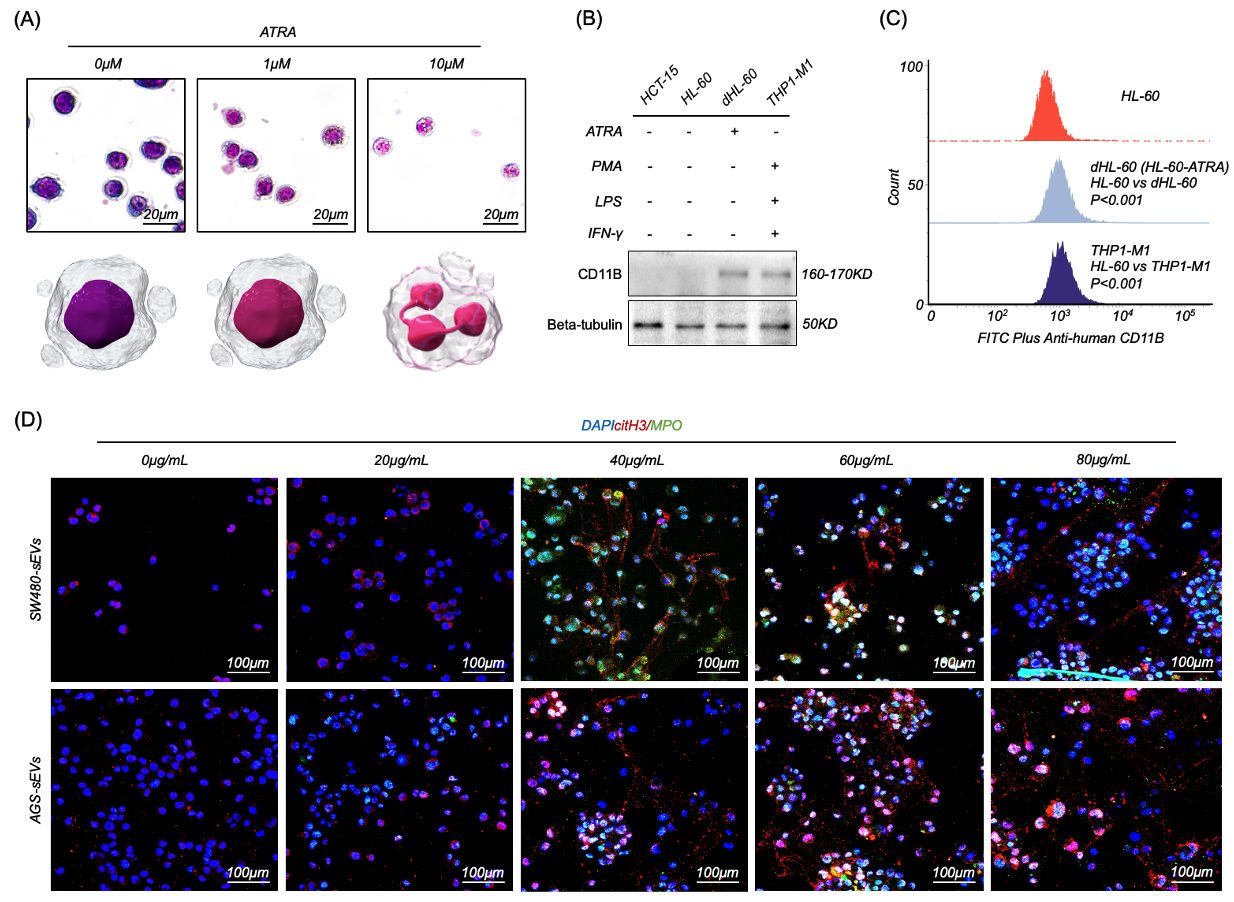
***

**3. Fig. S2. Analysis of protein concentrations of sEVs and lEVs in conditioned media from homologous cells.** (A) Protein concentrations of sEVs and lEVs in conditioned media from AGS. (B) Protein concentrations of sEVs and lEVs in conditioned media from SW480. Statistical results are presented as mean ± standard deviation (SD); **P*<0.05; ***P*<0.01; ****P*<0.001; *****P*<0.0001.

***
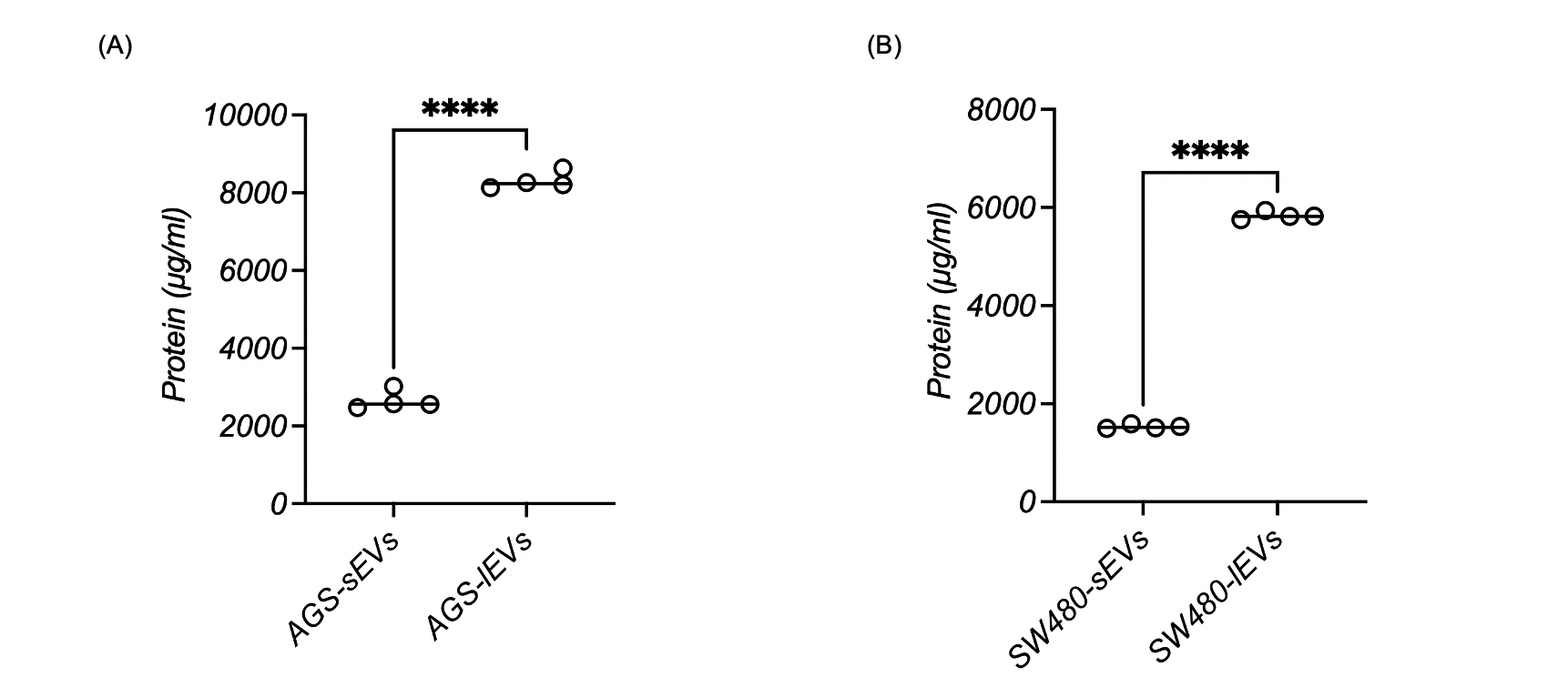
***

**4. Fig. S3. Exploration of positive control (PMA) concentration.** Induction of NETosis in dHL-60 by PMA at different concentrations. Statistical results are presented as mean ± standard deviation (SD); **P*<0.05; ***P*<0.01; ****P*<0.001; *****P*<0.0001.

***
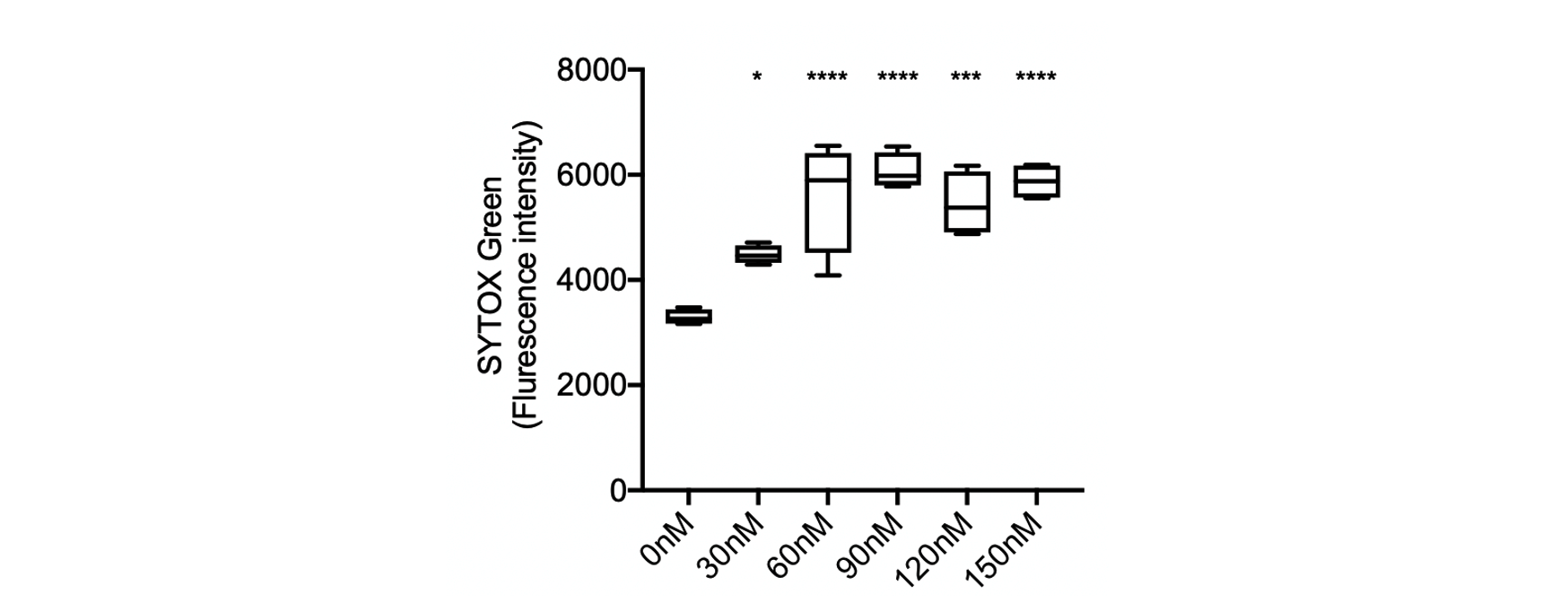
***

**
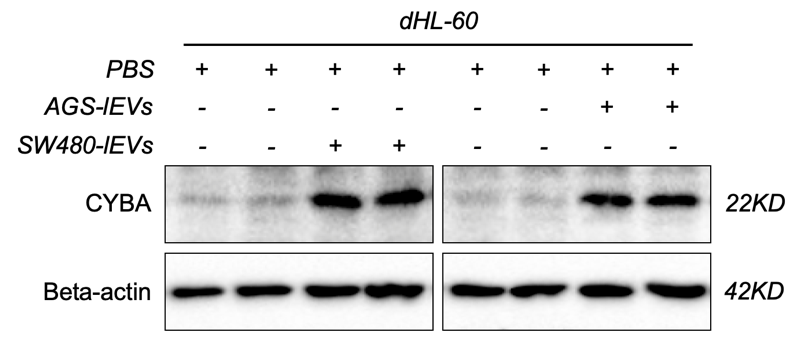
5. Fig. S4. Validation of tumor-derived lEVs delivering CYBA to dHL-60.**

**6. Fig. S5. Single-cell transcriptomics reveal *CYBA* may be a key effector molecule in NETosis-mediated VTE.** (A) DotPlot visualization of cell population annotations based on cluster-specific marker gene expression profiles. (B) Violin plots of *CYBA* expression in the normal, cancer and thrombosis groups. (C) GO enrichment analysis of differentially expressed genes (DEGs) in the thrombosis versus cancer comparison revealed significant enrichment of biological processes related to NETosis. (D) *CYBA* showed significant enrichment in ROS-related metabolic pathways among thrombosis-versus-cancer DEGs.

**
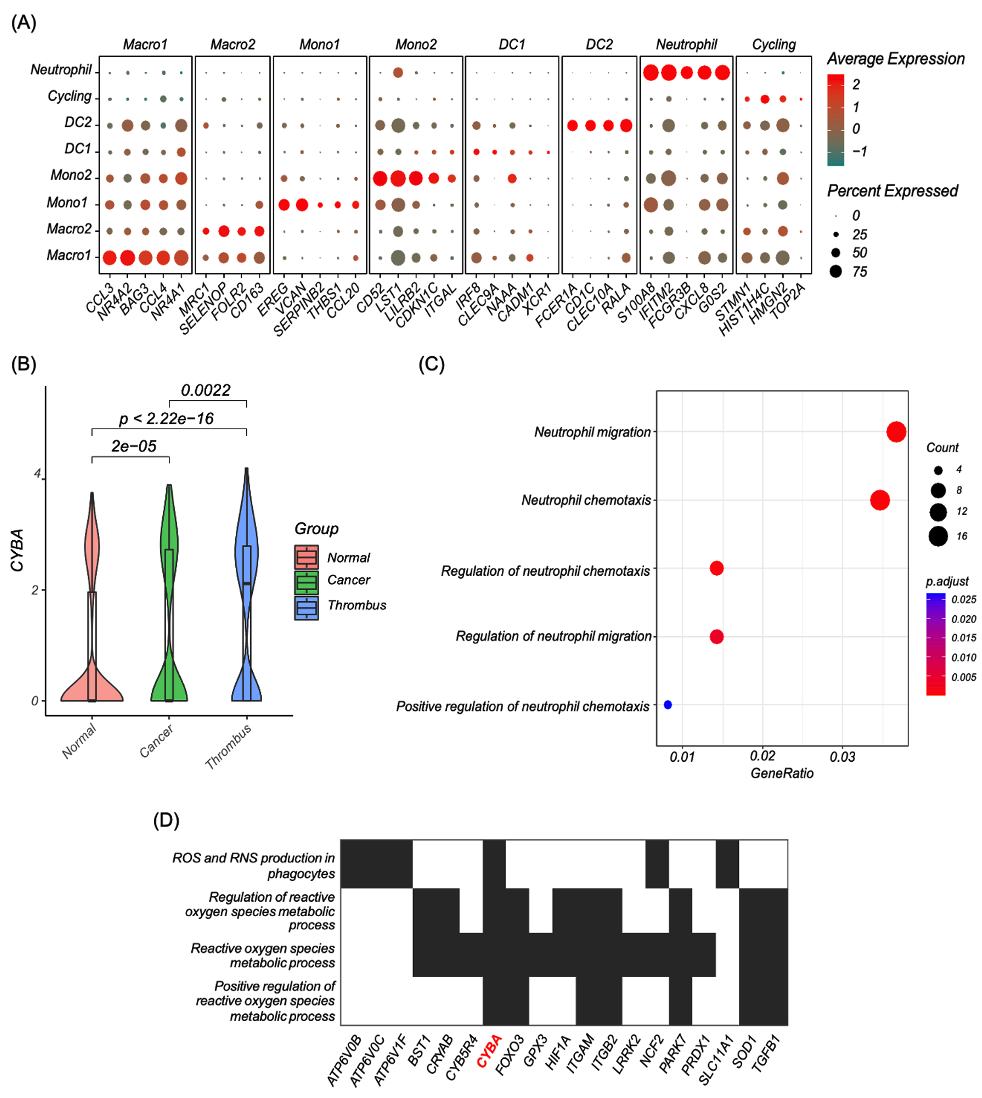
**

**7. Fig. S6. Validation of CYBA overexpression in AGS and SW480 and their lEVs.** (A-B) Validation of CYBA overexpression in AGS and SW480 cells. (C) Validation of CYBA overexpression in lEVs derived from cancer cells.

***
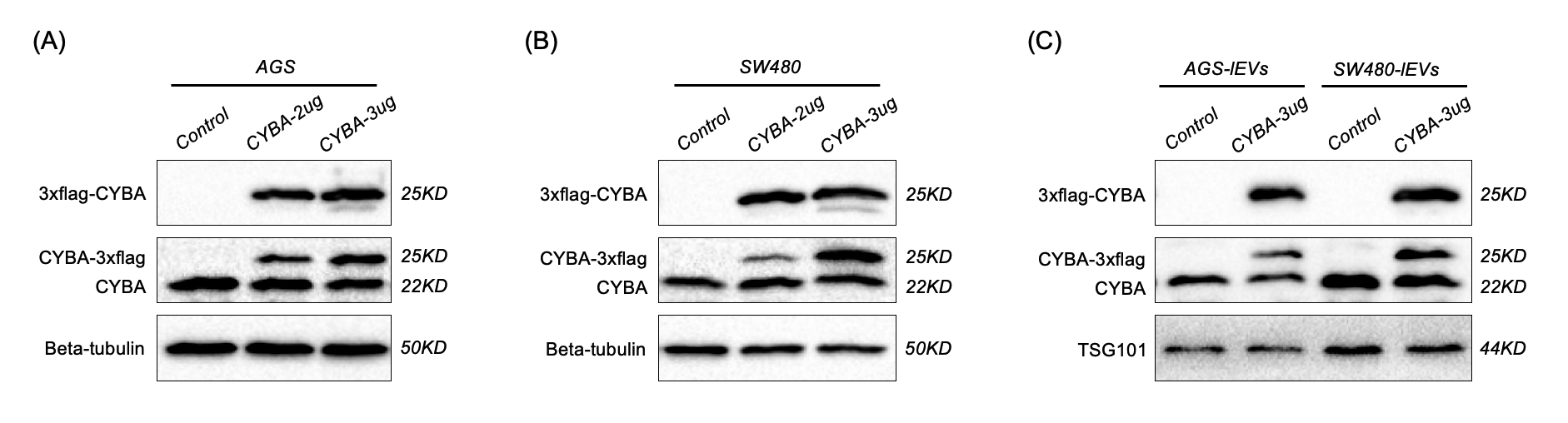
***

**8. Fig. S7. CYBA may not be a major contributing factor to exacerbation of NETosis induced by CC-lEVs.** (A) Confocal microscopy imaging of NETosis in dHL-60 following treatment with CYBA-overexpression CC-lEVs. (B-C) Flow cytometric anlaysis of ROS level in dHL-60 treated with CYBA-overexpression CC-lEVs. (D) WB analysis of citH3 expression in dHL-60 following treatment with CYBA-overexpression CC-lEVs. Statistical results are presented as mean ± standard deviation (SD); **P*<0.05; ***P*<0.01; ****P*<0.001; *****P*<0.0001.

***
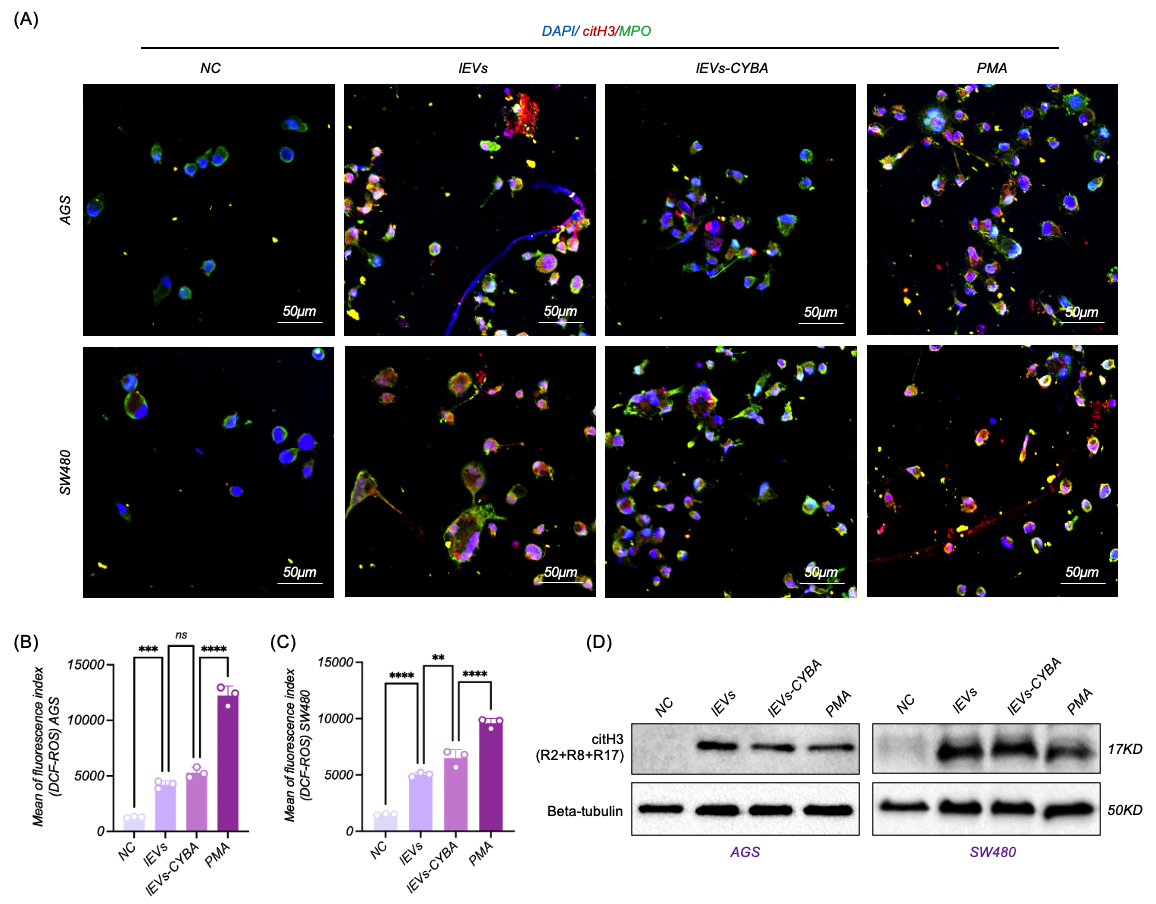
***

**9. Fig. S8. Analysis of *CYBA* biological function.** GeneMANIA report of *CYBA*.

***
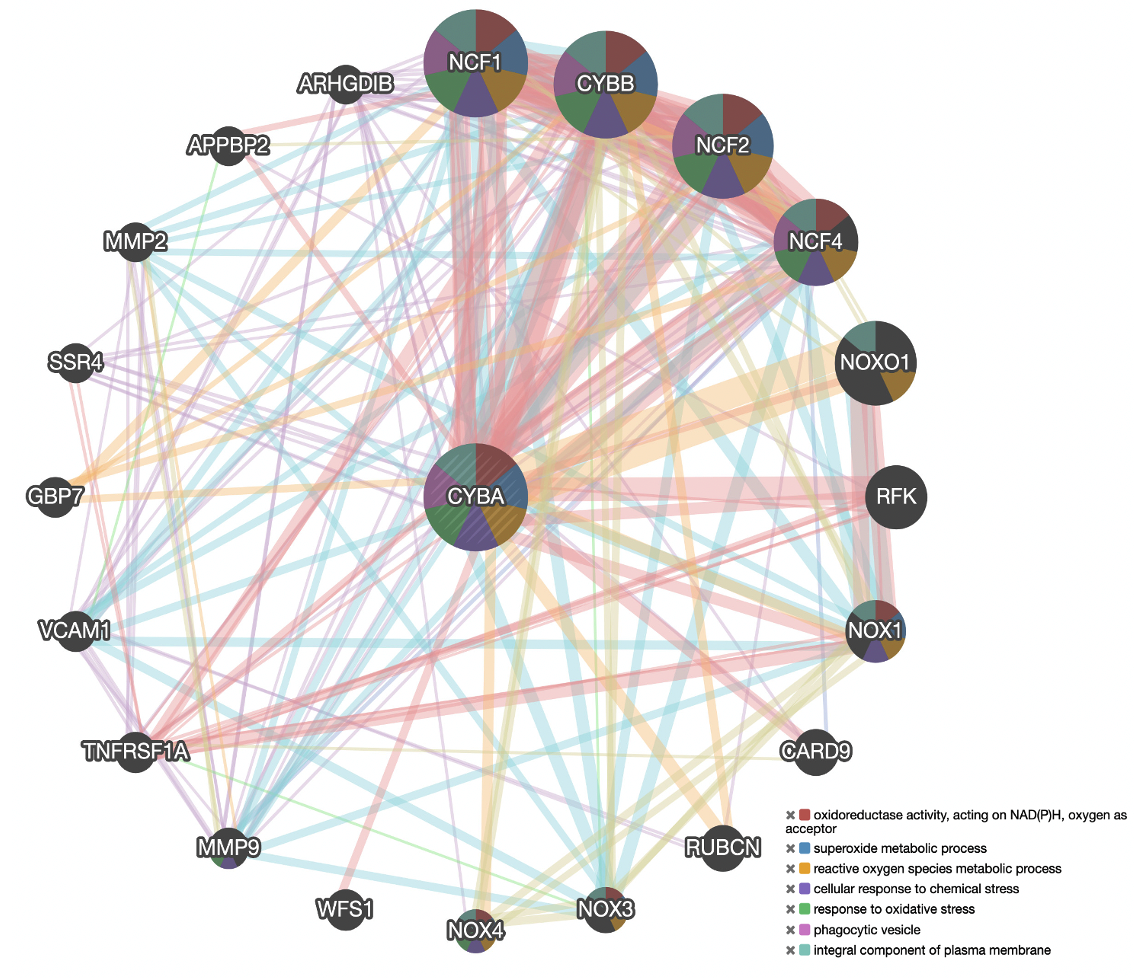
***

**10. Fig. S9. MiRNAs negatively correlated with *PAI-1 (SERPINE1)* expression in TCGA-STAD.** Statistical results are presented as mean ± standard deviation (SD); **P*<0.05; ***P*<0.01; ****P*<0.001; *****P*<0.0001.

***
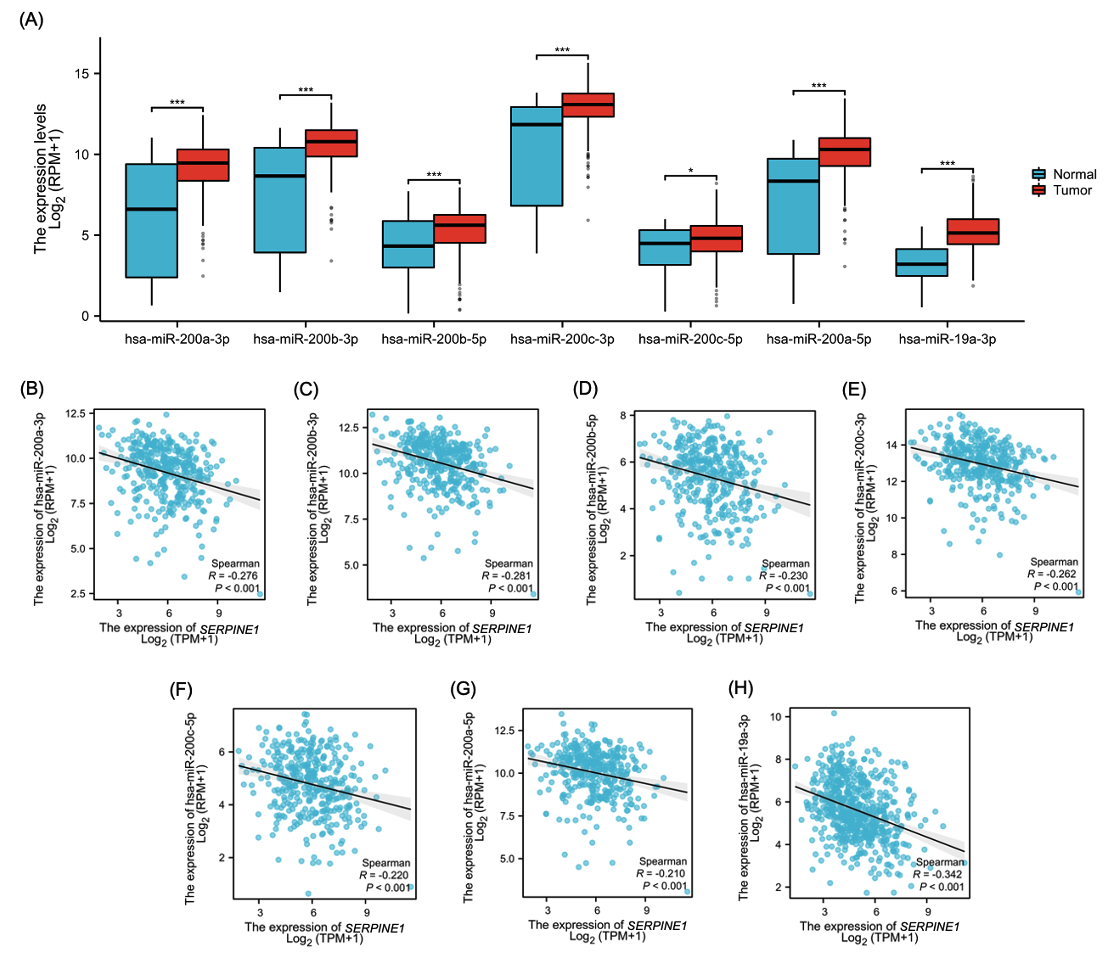
***

**11. Fig. S10. MiRNAs negatively correlated with *PAI-1 (SERPINE1)* expression in TCGA-COAD and TCGA-READ.** Statistical results are presented as mean ± standard deviation (SD); **P*<0.05; ***P*<0.01; ****P*<0.001; *****P*<0.0001.

***
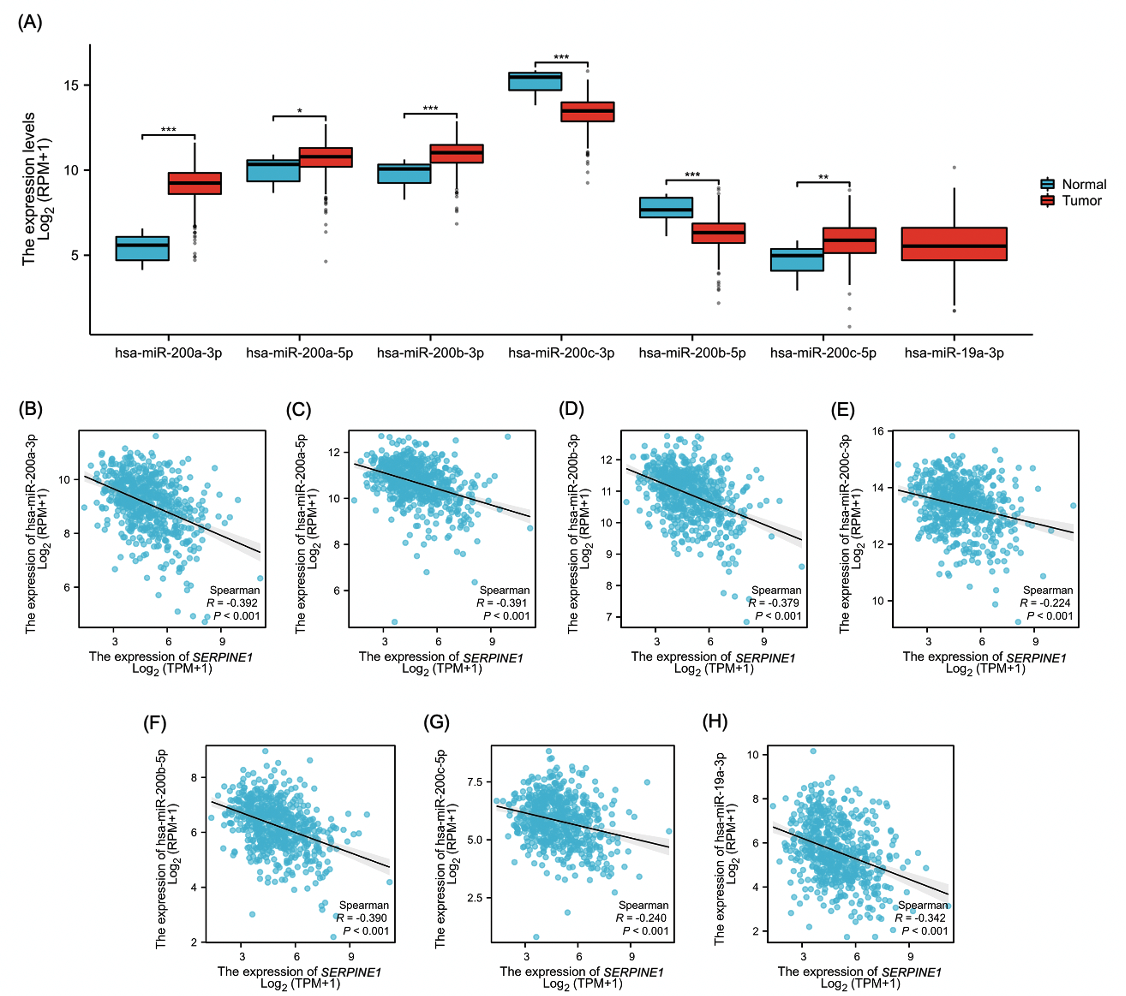
***

**12. Complete membrane and Uncropped bands of Fig. 1C**

**
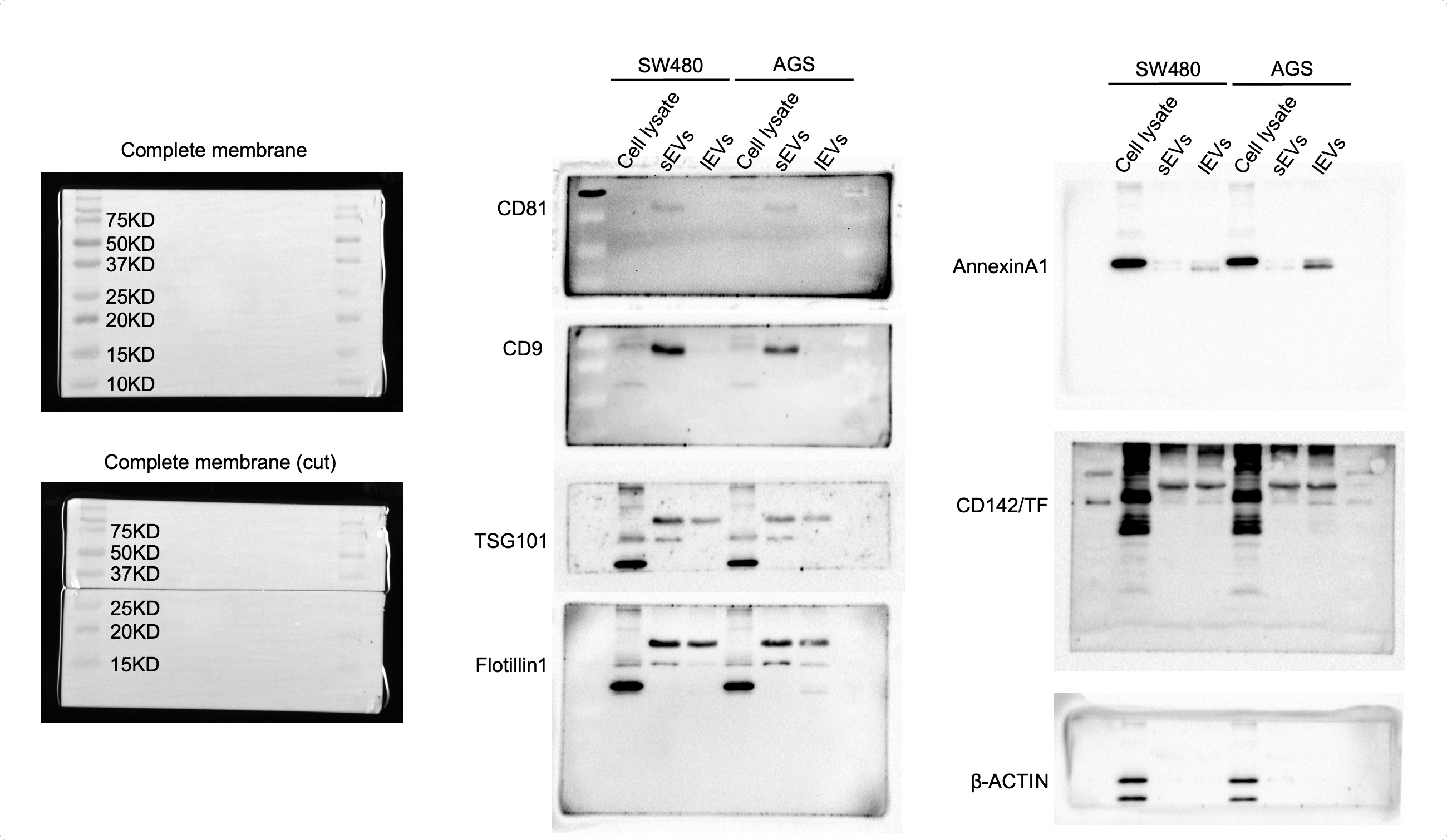
**

**13. Complete membrane and Uncropped bands of Fig. 1J**

**
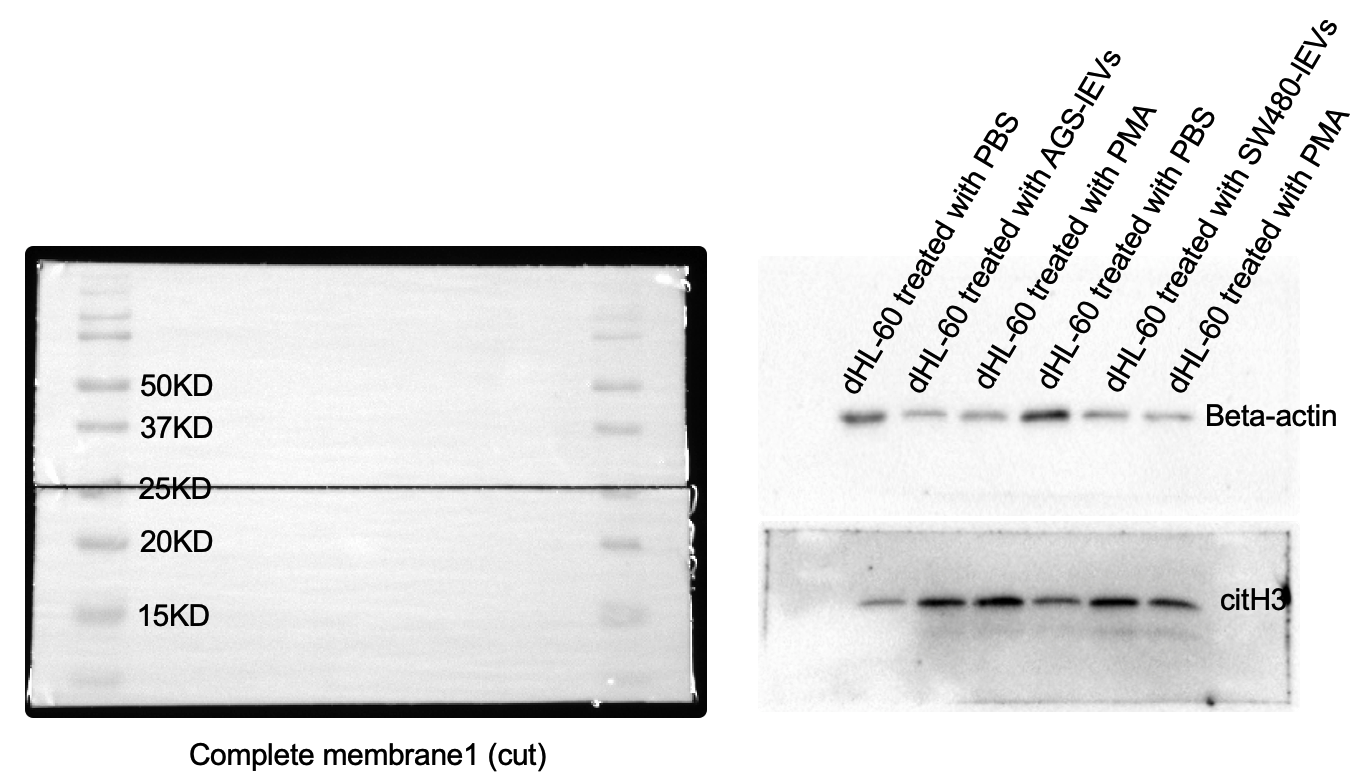
**

**
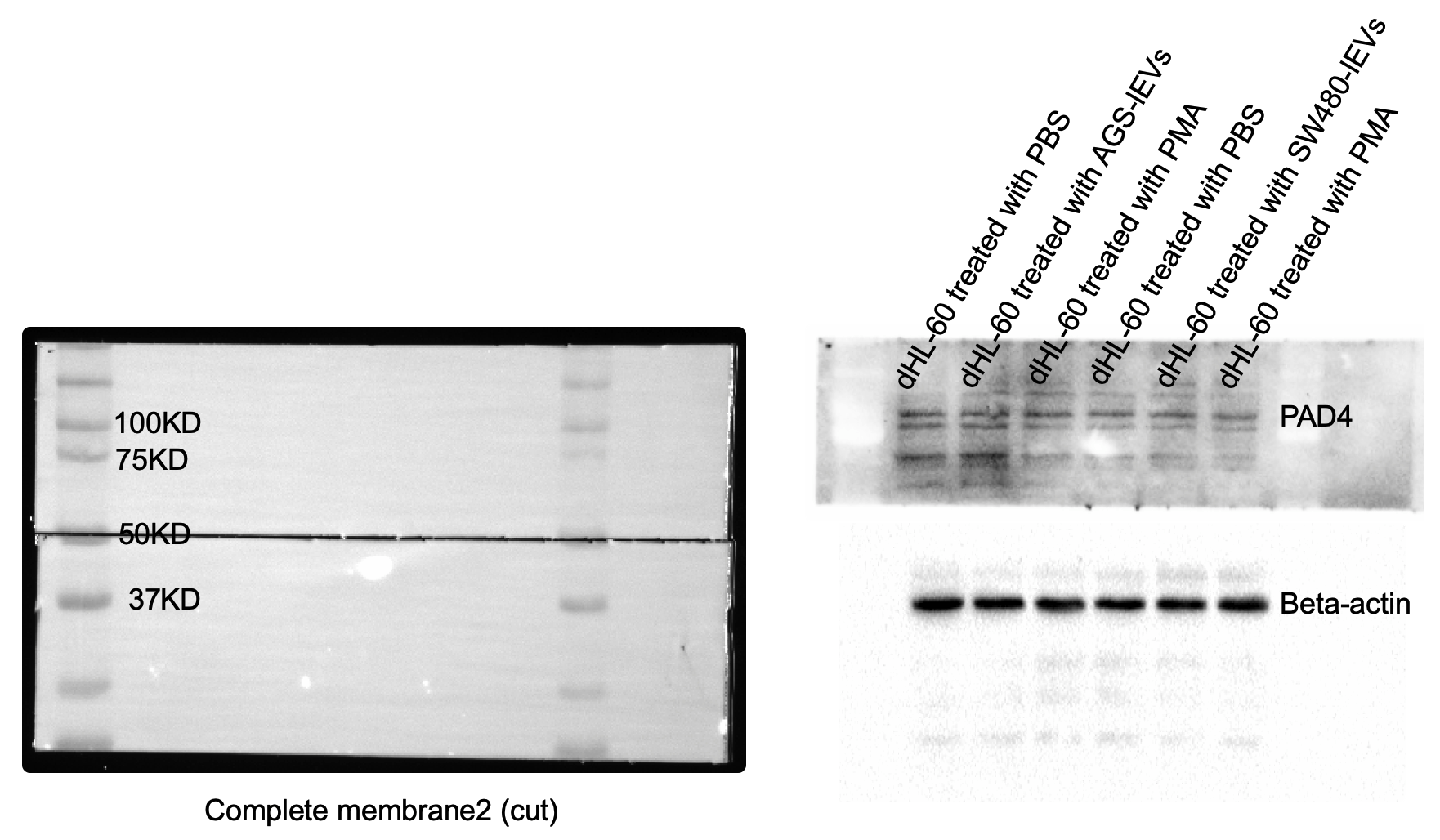
**

**14. Complete membrane and Uncropped bands of Fig. 2G**

**
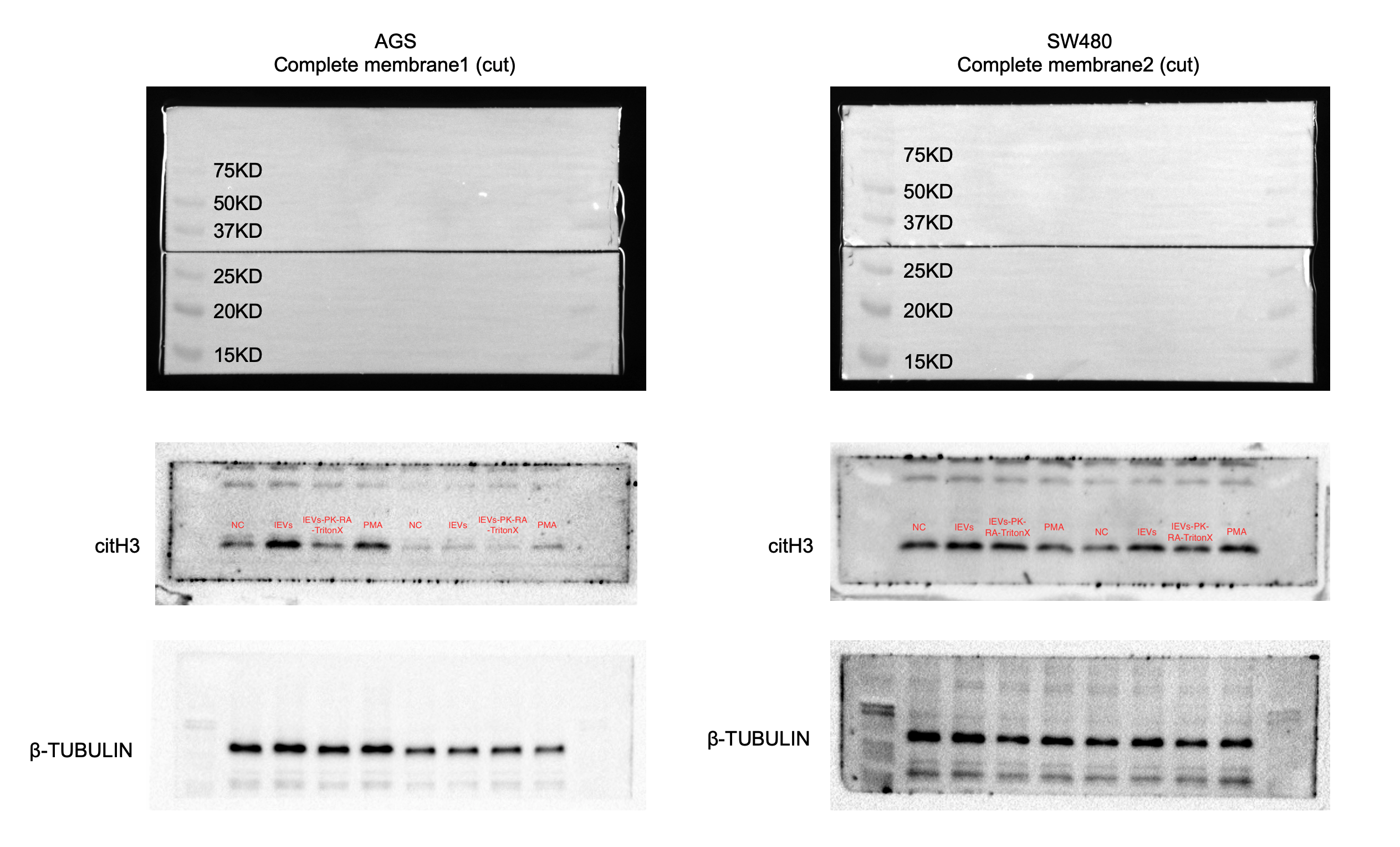
**

**15. Complete membrane and Uncropped bands of Fig. 3D**

**
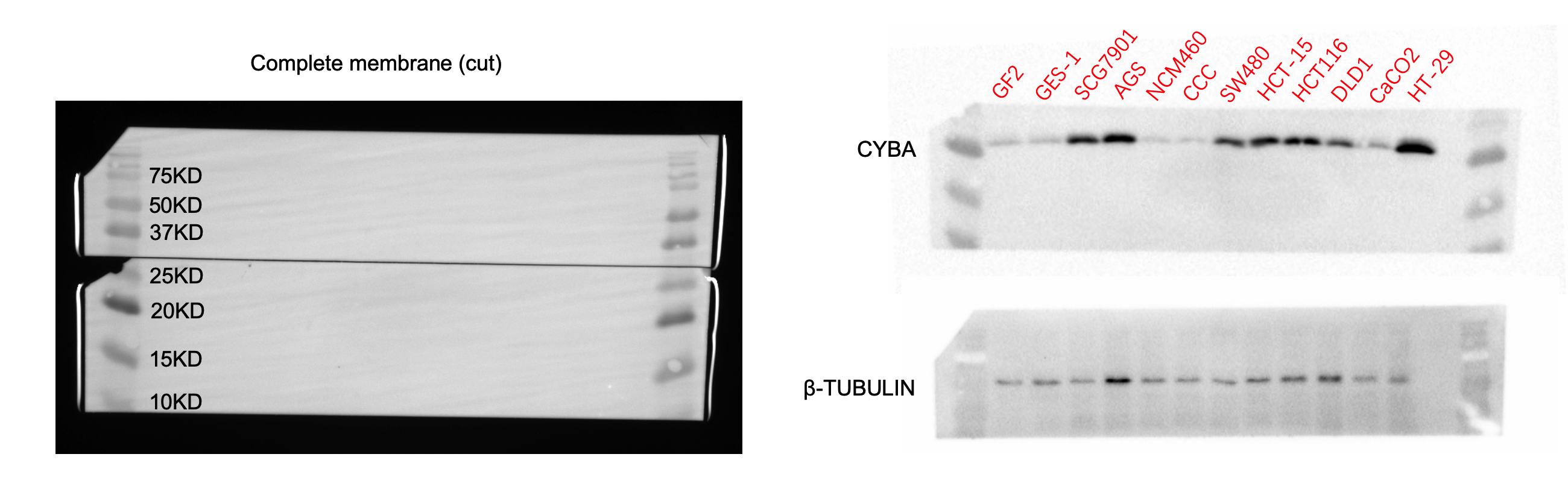
**

**16. Complete membrane and Uncropped bands of Fig. 3J**

**
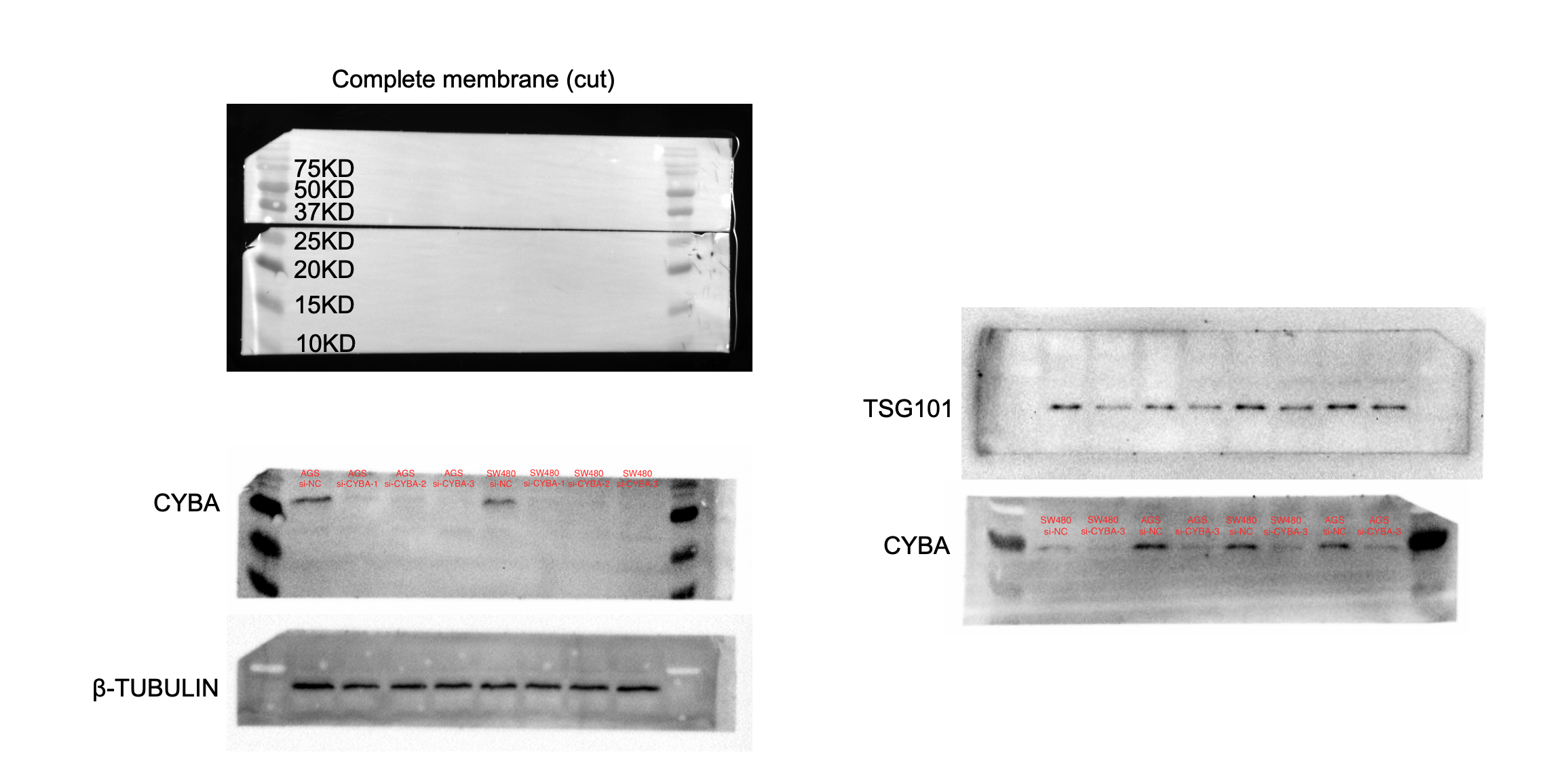
**

**17. Complete membrane and Uncropped bands of Fig. 3L**

**
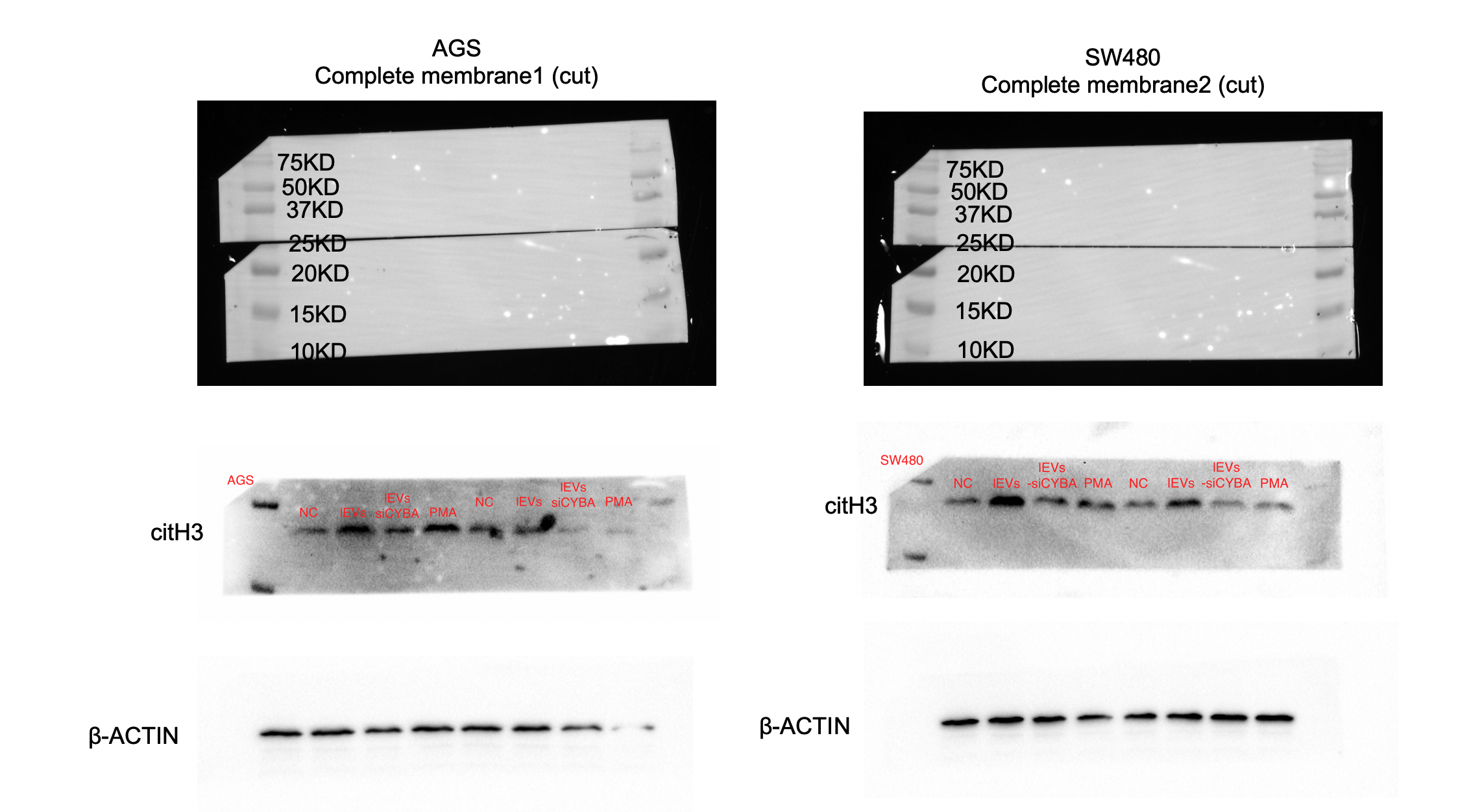
**

**18. Complete membrane and Uncropped bands of Fig. 4F**

**
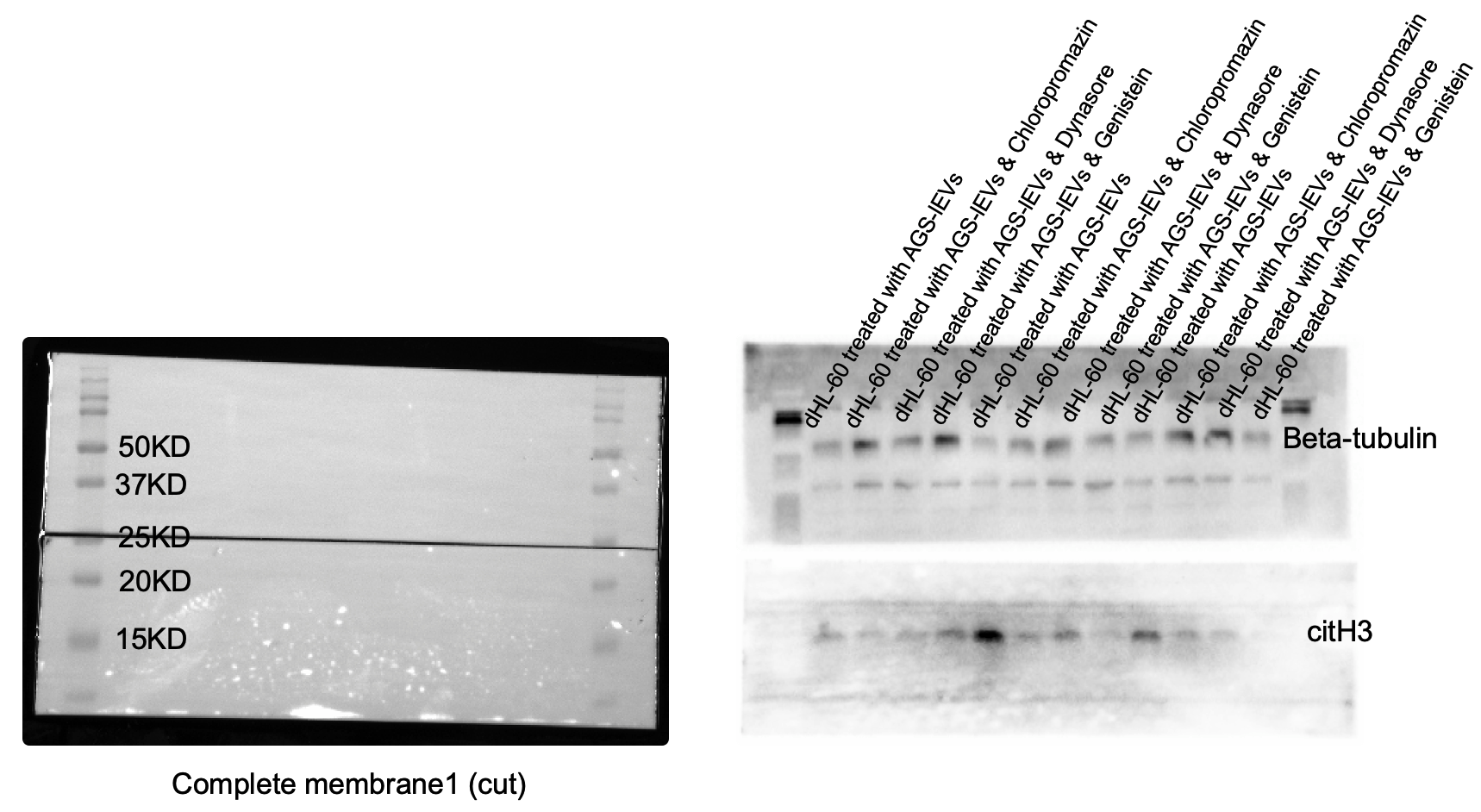
**

**
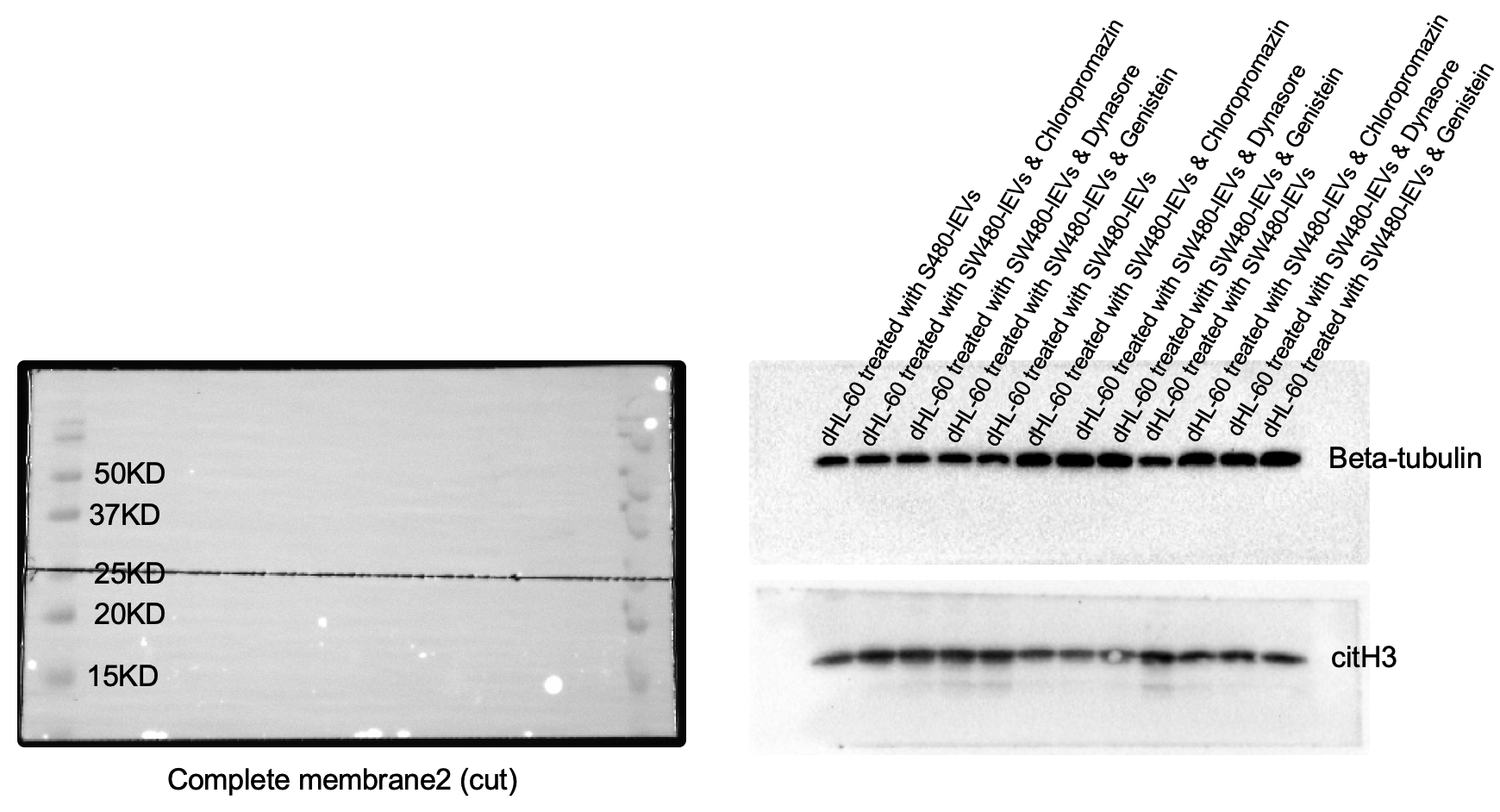
**

**19. Complete membrane and Uncropped bands of Fig. 5C**

**
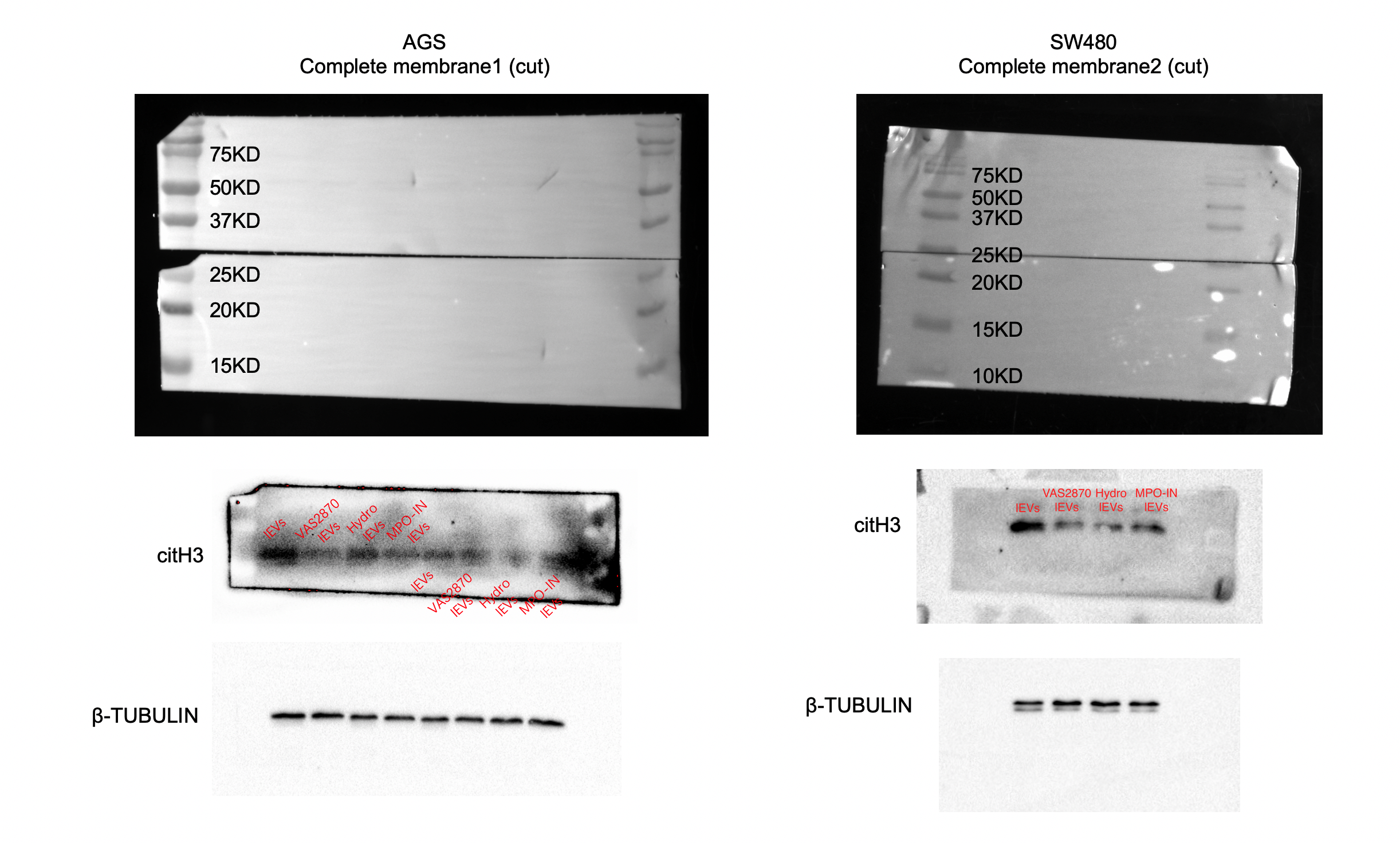
**

**20. Complete membrane and Uncropped bands of Fig. 6C**

**
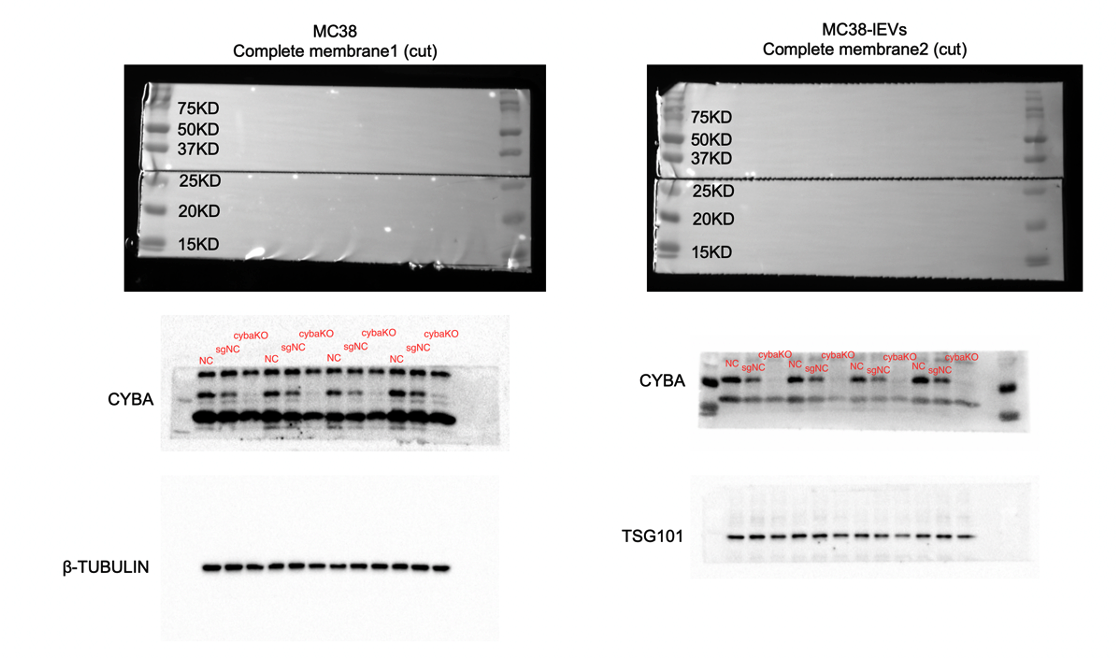
**

**21. Complete membrane and Uncropped bands of Fig. S1B**

**
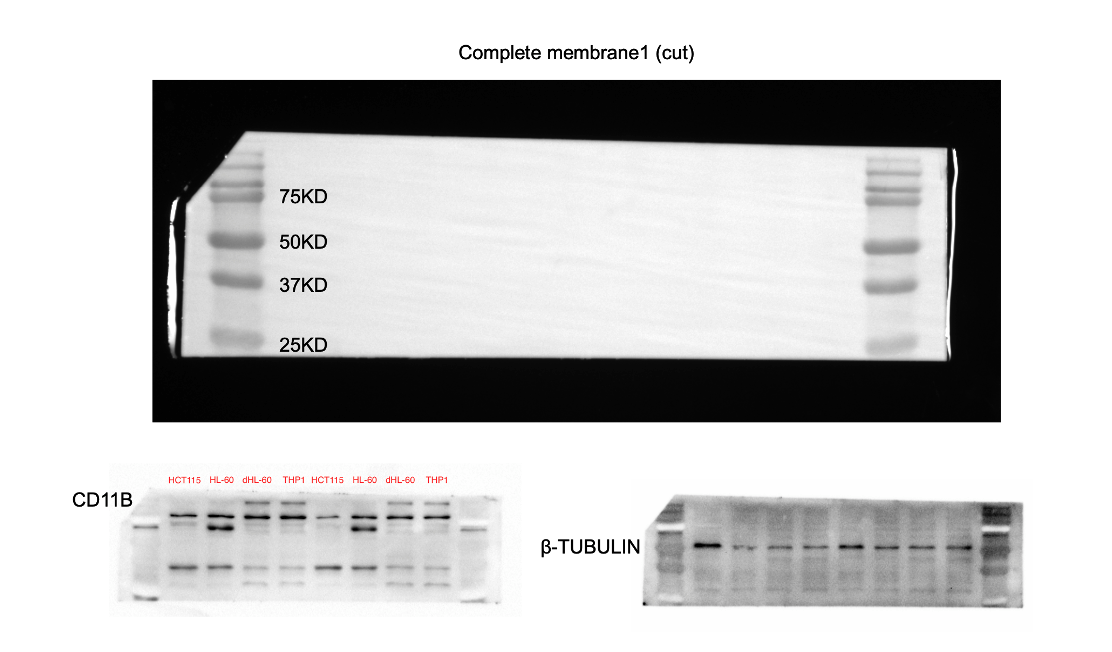
**

**22. Complete membrane and Uncropped bands of Fig. S4**

**
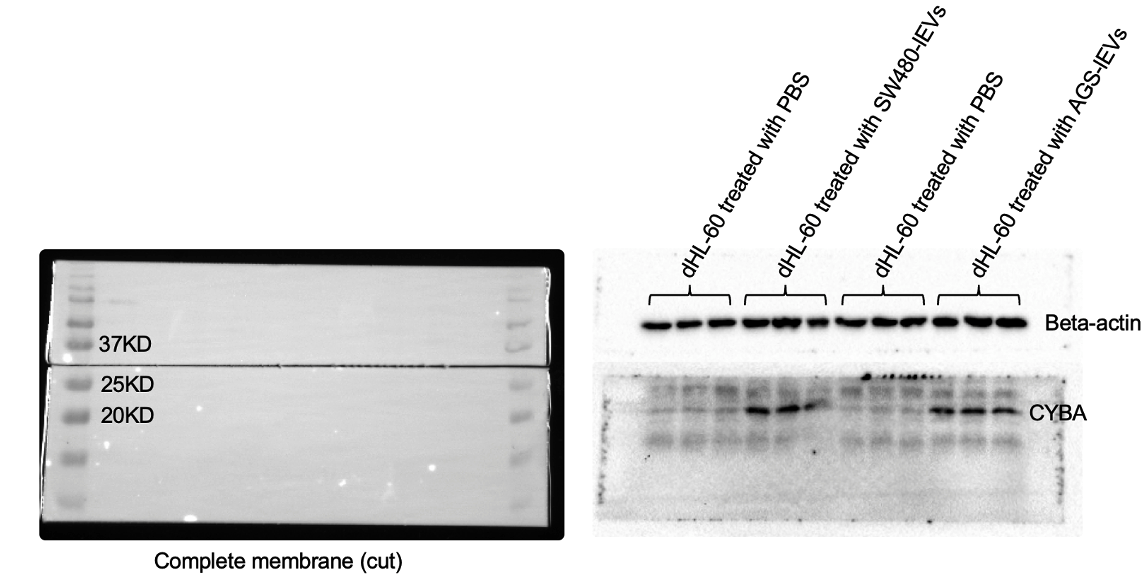
**

**23. Complete membrane and Uncropped bands of Fig. S6**

**
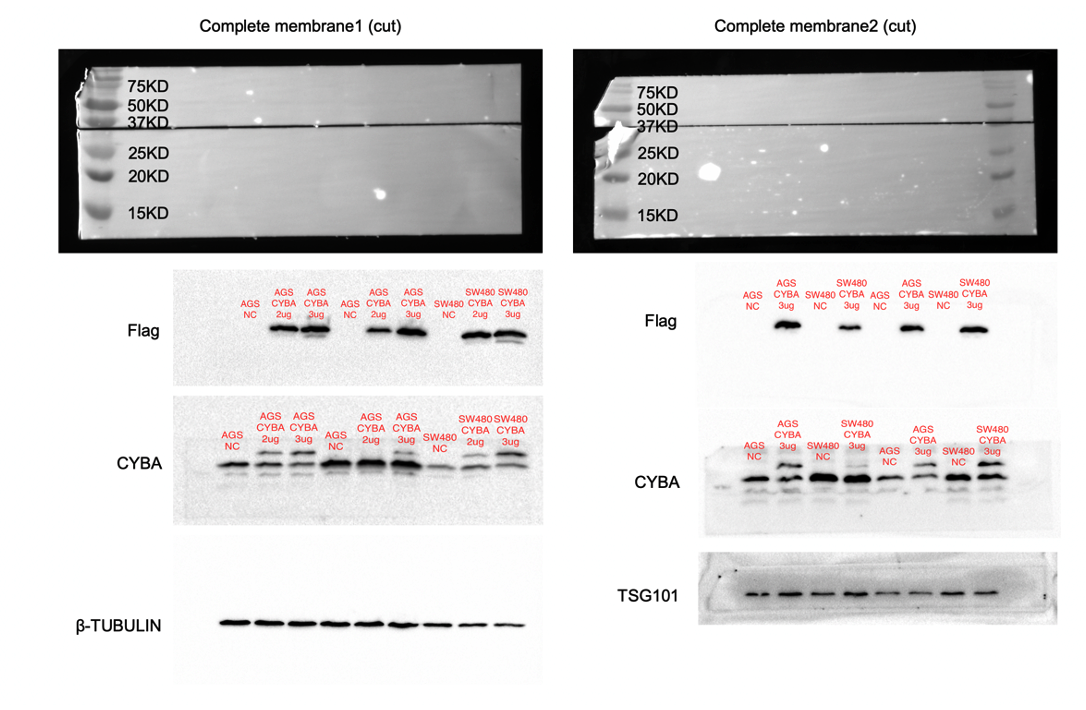
**

**24. Complete membrane and Uncropped bands of Fig. S7D**

**
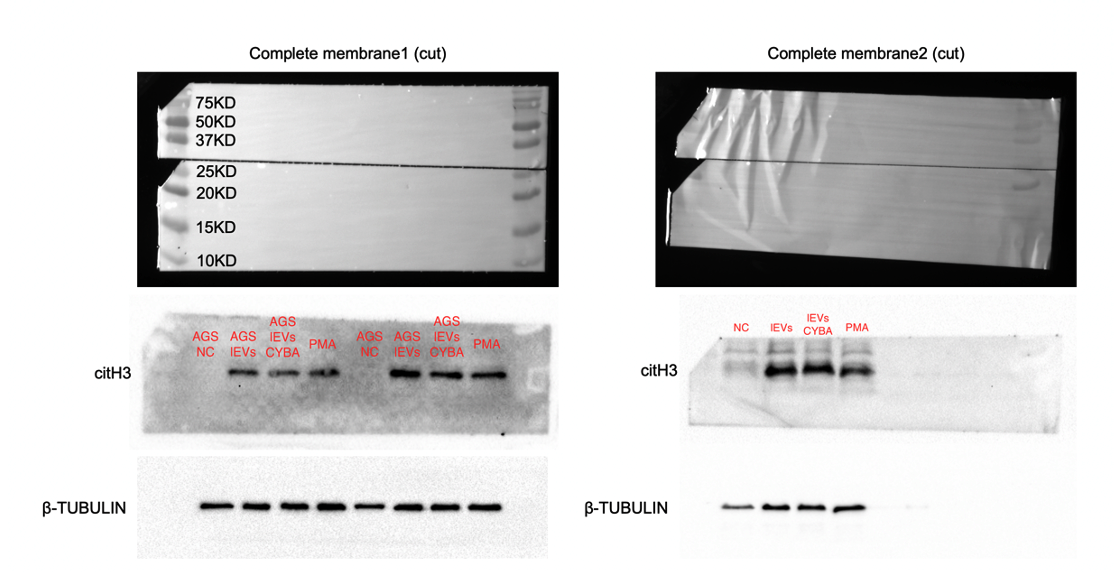
**
